# mlf-core: a framework for deterministic machine learning

**DOI:** 10.1093/bioinformatics/btad164

**Published:** 2023-04-02

**Authors:** Lukas Heumos, Philipp Ehmele, Luis Kuhn Cuellar, Kevin Menden, Edmund Miller, Steffen Lemke, Gisela Gabernet, Sven Nahnsen

**Affiliations:** Quantitative Biology Center (QBiC), Eberhard Karls University of Tübingen, Tübingen 72076, Germany; Institute of Computational Biology, Helmholtz Zentrum München, Munich 85764, Germany; Institute of Lung Biology and Disease and Comprehensive Pneumology Center, Helmholtz Zentrum München, Member of the German Center for Lung Research (DZL), Munich 81377, Germany; TUM School of Life Sciences Weihenstephan, Technical University of Munich, Freising 85354, Germany; Department of Informatics, University of Hamburg, Hamburg 20146, Germany; Quantitative Biology Center (QBiC), Eberhard Karls University of Tübingen, Tübingen 72076, Germany; Quantitative Biology Center (QBiC), Eberhard Karls University of Tübingen, Tübingen 72076, Germany; Department of Biological Sciences and Center for Systems Biology, The University of Texas at Dallas, Richardson, TX 75205, United States; Quantitative Biology Center (QBiC), Eberhard Karls University of Tübingen, Tübingen 72076, Germany; Quantitative Biology Center (QBiC), Eberhard Karls University of Tübingen, Tübingen 72076, Germany; Quantitative Biology Center (QBiC), Eberhard Karls University of Tübingen, Tübingen 72076, Germany; Biomedical Data Science, Department for Computer Science, Eberhard Karls University of Tübingen, Tübingen 72074, Germany; Institute of Bioinformatics and Medical Informatics, Eberhard Karls University of Tübingen, Tübingen 72074, Germany; Faculty of Medicine, Eberhard Karls University of Tübingen, Tübingen 72016, Germany

## Abstract

**Motivation:**

Machine learning has shown extensive growth in recent years and is now routinely applied to sensitive areas. To allow appropriate verification of predictive models before deployment, models must be deterministic. Solely fixing all random seeds is not sufficient for deterministic machine learning, as major machine learning libraries default to the usage of nondeterministic algorithms based on atomic operations.

**Results:**

Various machine learning libraries released deterministic counterparts to the nondeterministic algorithms. We evaluated the effect of these algorithms on determinism and runtime. Based on these results, we formulated a set of requirements for deterministic machine learning and developed a new software solution, the mlf-core ecosystem, which aids machine learning projects to meet and keep these requirements. We applied mlf-core to develop deterministic models in various biomedical fields including a single-cell autoencoder with TensorFlow, a PyTorch-based U-Net model for liver-tumor segmentation in computed tomography scans, and a liver cancer classifier based on gene expression profiles with XGBoost.

**Availability and implementation:**

The complete data together with the implementations of the mlf-core ecosystem and use case models are available at https://github.com/mlf-core.

## 1 Introduction

In recent years, machine learning (ML) has seen applications in almost all areas of the sciences and impacts society even in hidden ways, such as in the assessment of loan eligibility (Kruppa et al. 2013), clinical decision support (Beam and Kohane 2018), and crime or terrorist detection (Tayal et al. 2015). To adhere to democratic transparency standards and due to the sensitive application areas, reproducibility has been identified as a key factor to consider when applying ML (Hutson 2018; Heil et al. 2021). Reproducibility in ML was defined by Gunderson and Kjensmo as “the ability of an independent research team to produce the same results using the same artificial intelligence (AI) method based on the documentation made by the original team” (Gundersen and Kjensmo 2018).

Although the field of ML is progressing at a rapid pace, not all findings can be verified and reproduced (Olorisade et al. 2017; Hutson 2018). Collberg and Proebsting (2016) evaluated 402 computational experimental papers and could only reproduce 48.3% even when communicating with the authors (Wilkinson et al. 2016). Gunderson and Kjensmo report that for AI papers “only between a fifth and a third of the variables required for reproducibility are documented” (Gundersen and Kjensmo 2018). The reasons for irreproducible ML are diverse, including unavailable (raw) data, unpublished code, unreported hyperparameters, and missing reports on the hardware and software used for the analysis (Olorisade et al. 2017; Henderson et al. 2018; Haibe-Kains et al. 2020).

We argue that determinism should be equally considered in ML. Deterministic ML can be defined as the ability of an independent researcher to reproduce the *bit-exact* same results based on the complete documentation by the original team together with the model code when run on the same computational infrastructure.

Deterministic models have advantages beyond reproducibility which allow reproducing the exact same model metrics and predictions when the same training data are provided, and ease debugging by accurately describing metrics and predictions without the influence of unknown random factors. Moreover, they enable targeted experimentation, where the model performance cannot be attributed to random seeds. Scientists, developers, and the general public benefit from deterministic ML, as a step towards building trustworthy ML (Toreini et al. 2020).

Recently, several papers have compiled the requirements for the reproducibility of ML algorithms and provided reproducibility checklists (Mongan et al. 2020; Norgeot et al. 2020; Heil et al. 2021; Matschinske et al. 2021). Even when adhering to these checklists, and reporting the utilized hardware and software together with the corresponding code, deterministic models are not necessarily obtained. This is especially relevant when the models are trained on graphical processing units (GPUs), which employ nondeterministic functions based on atomic operations (Nagarajan et al. 2019). Several ML frameworks exist that aim at solving the reproducibility issue. Projects such as Guild AI (https://guild.ai/) and Sacred (https://github.com/IDSIA/sacred) allow tracking all performed experiments and parameters used, and visualize those on a dashboard. Polyaxon (https://polyaxon.com/) and MLFlow (https://www.mlflow.org) additionally introduce containerization to ensure that the same software environment is used at runtime (Table 1). However, none of the ML experiment tracking frameworks currently track the hardware employed for the analysis or ensure that only deterministic functions are employed during training and inference. Therefore, there is a need for a single, user-friendly framework incorporating all requirements for deterministic ML.

**Table 1.** Comparison between different ML experiment tracking frameworks.

Framework	Tracking	Visualization	Container	Hardware	Determinism
Polyaxon	Full	Dashboard	Docker	No	No
Guild AI	Full	Dashboard	No	No	No
Sacred	Full with dependencies	Dashboard	No	Limited	No
MLflow	Full with models	Dashboard	No	No	No

Tracking includes capabilities of monitoring experiments, hyperparameters, metrics, artifacts, models, or software dependencies. Visualization denotes the possibility of visually keeping track of experiments and automatic plotting abilities. Containers allow for consistent runtime environments. Hardware describes the competence of tracking the used hardware (model, quantity, and generation) for any run. Sacred tracks the available hardware, but not solely the used hardware. Determinism describes the ability to statically verify that the code is deterministic.

Here, we present mlf-core, an ML framework that enables building fully deterministic and therefore also reproducible ML projects. mlf-core is based on MLflow for ML experiment tracking, visualization, and model deployment. Additionally, mlf-core provides project templates and static code analysis (linting) functionality that ensures the sole usage of deterministic algorithms for GPU computing as well as setting all necessary random seeds for deterministic results. We identified the necessary settings to ensure determinism by analyzing the hardware- and software-derived sources of nondeterminism in three ML models built with the PyTorch (Paszke et al. 2019), TensorFlow (Abadi et al. 2015), and XGBoost (Chen and Guestrin 2016) libraries. We concluded that setting random seeds alone was not sufficient to ensure determinism in GPU-based computations. When enabling the full deterministic requirements, deterministic results were obtained for all models.

We showcase the applicability of mlf-core to three use cases for various biomedical applications. First, using an autoencoder model built with TensorFlow, we demonstrate how nondeterministic model training and inference can strongly influence the outcome of algorithms commonly used to analyze single-cell RNA-sequencing (scRNA-seq) data. Second, we exhibit how feature importance varies for nondeterministic runs with a liver cancer classifier based on transcriptomics data implemented with XGBoost. Third, we implemented a U-Net (Çiçek et al. 2016) model with Pytorch for liver-tumor segmentation in abdominal computed tomography (CT) scans showing that only deterministic training yields the exact same model predictions.

Together with providing access to FAIR (Findable, Accessible, Interoperable, and Reusable) (Wilkinson et al. 2016) training data and code, mlf-core enables researchers to perform deterministic ML research.

## 2 Materials and methods

### 2.1 mlf-core ecosystem implementation

The mlf-core ecosystem comprises the two Python packages mlf-core (https://github.com/mlf-core/mlf-core) and system-intelligence (https://github.com/mlf-core/system-intelligence), a collection of GPU-enabled Docker containers (https://github.com/mlf-core/containers), and the corresponding website (https://mlf-core.com).

The mlf-core and system-intelligence Python packages are based on the nf-core (Ewels et al. 2020) project, and cookietemple’s cli-python template (Lukas Heumos et al. 2020). The creation of the ML templates is primarily facilitated by cookiecutter (https://github.com/cookiecutter/cookiecutter) to replace variable defaults with user-defined choices. The templates themselves are based on the determinism experiments conducted in this work, extended with MLflow (https://mlflow.org) integration.

All mlf-core templates are versioned allowing for the comparison of existing project’s former template versions and the latest mlf-core release’s template version. If newer versions are available a sync pull request incorporating all new changes is created against the existing project. This process uses the user’s GitHub account together with the user’s GitHub Token which was securely saved as a GitHub secret during the project creation step. In the same step, a *TEMPLATE* branch is created, which at any time only contains the template code. When a template sync occurs, the project template is recreated on the *TEMPLATE* branch and a pull request based on the new template is submitted against the development branch. A GitHub Actions-based workflow runs on a daily basis to check for new template versions.

The verification of best practices and ML determinism is facilitated through a custom static code analyzer, which checks the complete code against predefined sets of deterministic functions and points out nondeterministic functions when they are used. These sets are continuously updated and new developments lead to new mlf-core releases. The static code analyzer runs on every push and pull request event against any branch on GitHub or can be called manually with the *mlf-core lint* command.

The base Docker container for all mlf-core template Docker container is *nvidia/cuda : 11.2.1-cudnn8-devel-ubuntu20.04* extended with Miniconda 4.9.2. Additionally, the PyTorch template defines the *CUBLAS_WORKSPACE_CONFIG=:4096:8* environment variable to disable the nondeterministic behavior of multi-stream execution in internal workspace selection for routines running in parallel streams.

### 2.2 Determinism evaluation

To evaluate determinism on the PyTorch, Tensorflow, and XGBoost libraries, three different experimental setups were employed to train:

- Random: no random seeds were set.
- Seeds: Random seeds were set for NumPy, Python’s Random module, and the Python hash seed. Additionally, random seeds for the respective evaluated libraries—PyTorch, TensorFlow, and XGBoost were set.
- Deterministic: All random seeds of the setup *seeds* were set together with the enforcing of all deterministic algorithms (Supplementary Fig. S1). cuDNN benchmark was disabled as it can select nondeterministic functions. For the Tensorflow library, the *TF_DETERMINISTIC_OPS* environment variable was set. For the XGBoost library, the option *single_precision_histogram* was enabled, and XGBoost’s *allreduce* operations were avoided since they have not yet been verified to run deterministically. For the PyTorch library, all functions labeled as knowingly nondeterministic by PyTorch (https://pytorch.org/docs/stable/notes/randomness.html) were explicitly avoided.

Details on the exact library versions and models employed for the evaluation, as well as the use case model implementation can be found in the Supplementary Methods.

## 3 Results

### 3.1 The mlf-core ecosystem for deterministic ML

**Figure 1:**
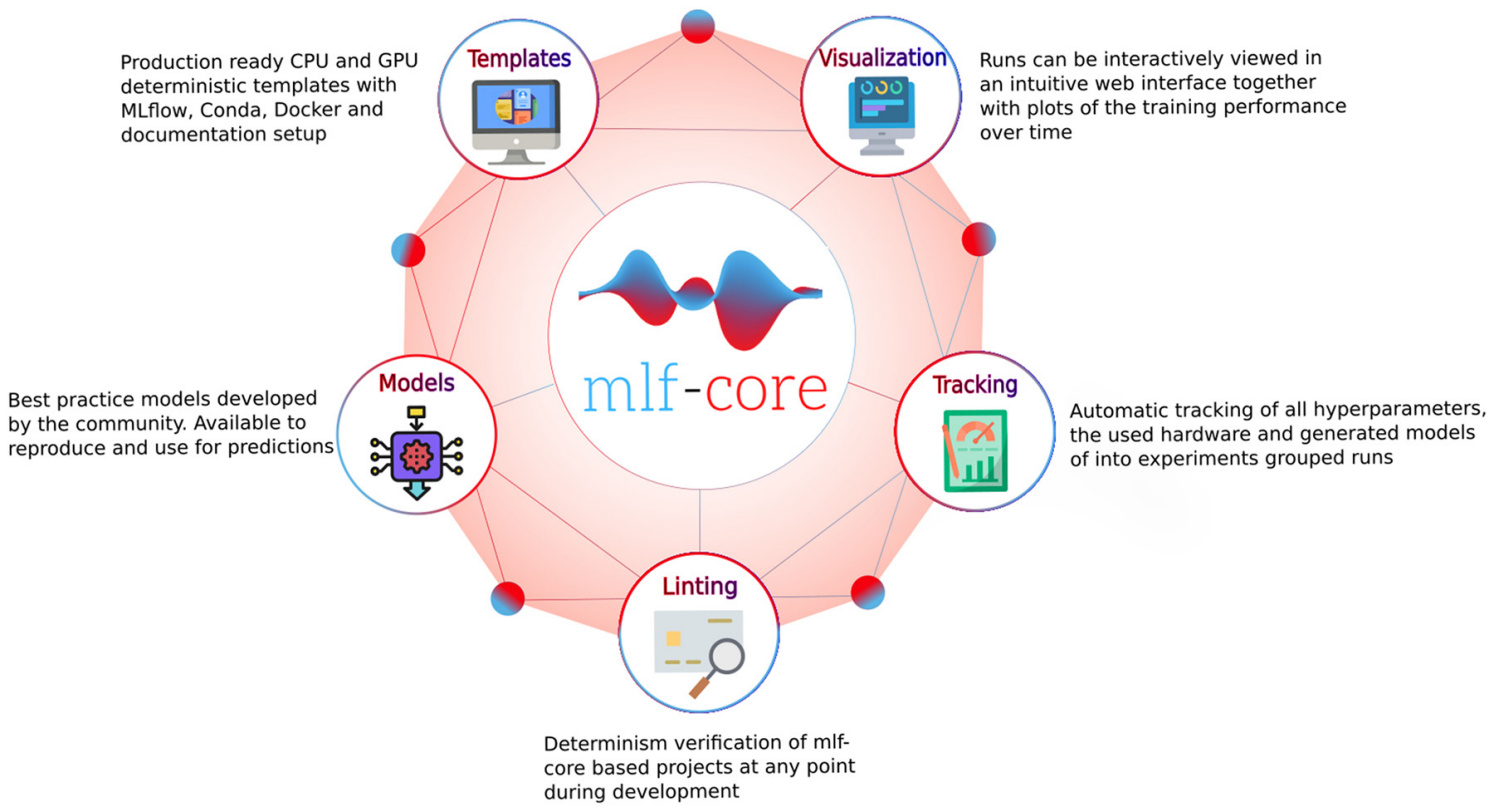
Overview of the mlf-core ecosystem. The mlf-core ecosystem comprises the Python packages mlf-core and system-intelligence, community developed reproducible mlf-core models and a set of GPU enabled Docker containers

To address the need for an ecosystem that enables the easy setup of deterministic ML models, we developed mlf-core. mlf-core is inspired by and based on nf-core (Ewels et al. 2020), a framework for building reproducible bioinformatics pipelines written in Nextflow; and cookietemple (Lukas Heumos et al. 2020), a collection of best-practice templates for various domains and programming languages. mlf-core provides a complete solution for deterministic ML that ensures deterministic model training and tackles other important aspects for determinism of the developed models, such as result visualization and hyperparameter tracking, operating system tracking, dependency management with containers, and a publicly hosted model documentation (Fig. 1).

mlf-core allows for the interactive creation of deterministic ML projects based on best-practice templates for the most widely used ML frameworks, namely PyTorch, TensorFlow, or XGBoost. These templates include a minimal running example for training e.g. a convolutional neural network or a distributed gradient boosting model which meets a set of requirements that have been determined and empirically verified to enable deterministic ML model training on both the CPUs or GPUs. These requirements include the containerization of the runtime environment, the setting of all random seeds for all used libraries, the usage of solely deterministic algorithms, and the tracking of all hyperparameters and metrics.

**Figure 2:**
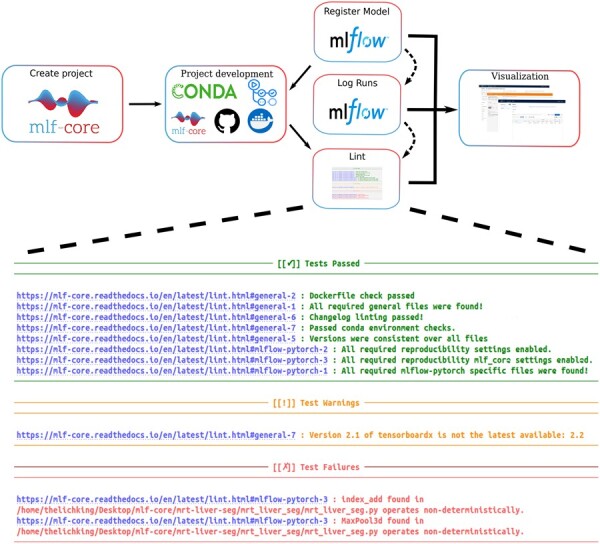
Workflow for the development of a mlf-core based model. Projects are created interactively with the mlf-core command line tool, and automatically published to a newly created GitHub repository. Project development is facilitated with the use of a continuous integration pipeline using GitHub Actions and isolated runtime environments based on Conda and Docker. mlf-core lint statically validates determinism and informs on violations. All training runs log all metrics and save models in a specified model registry. The MLflow web interface and Tensorboard facilitate interactive exploration of the generated models. A tutorial for the complete workflow is available at https://mlf-core.readthedocs.io/en/latest/tutorial.html

A custom code linter is provided as part of the mlf-core Python package that checks whether already developed projects adhere to the determined requirements for deterministic ML. If any setting in the code potentially leads to nondeterministic models (e.g. not all required random seeds are set, deterministic algorithms are not enforced, or known nondeterministic algorithms are used), the user is warned and possible deterministic alternatives are proposed (Fig. 2). Enabling and disabling determinism modes allows researchers to quantify the impact of nondeterministic training and prediction in downstream analysis, such as statistical significance evaluations.

Furthermore, the hardware used for training is automatically logged and reported with system intelligence, a tool of the mlf-core ecosystem, even in distributed environments with different hardware architectures. All mlf-core project templates include an MLflow setup to use Conda (Anaconda, 2016) or Docker (Merkel, 2014) containers for dependency management, which are recommended to ensure interoperability in a variety of infrastructures. Continuous integration workflows based on GitHub Actions (https://github.com/features/actions) enable the automatic building of the Docker container and statically verify code quality (linting). Sphinx (https://www.sphinx-doc.org) is employed for documentation building in order to encourage developers to document their ML project. The mlf-core project creation process ends with the option to automatically upload the model code to a newly created GitHub repository to encourage code sharing (Fig. 2). Existing projects are kept up to date with the mlf-core sync functionality (see Supplementary Methods) allowing for regular updates to ensure determinism and adherence to current best-practices. Already existing projects are easily transformed into mlf-core projects by solely copying the model code into the corresponding section in the template. To enable easy model scores and hyperparameter tracking, mlf-core employs MLflow to provide an overview of all the training runs and automatically log all hyperparameters and obtained metrics for each run as well as an instance of the trained model. The automatically saved Tensorboard events can be used to visualize the model graph and tensor trajectories (Supplementary Fig. S2).

mlf-core’s tight integration of MLflow also allows for models to be easily served as representational state transfer APIs, effectively bridging research and application by allowing users to request predictions of the model. Model training and inference can be performed in various infrastructures including all common Cloud providers (e.g. Amazon Web Services and Google Cloud) and scales to big data with Apache Spark (Zaharia et al. 2016).

### 3.2 Validation of ML determinism with mlf-core

**Figure 3:**
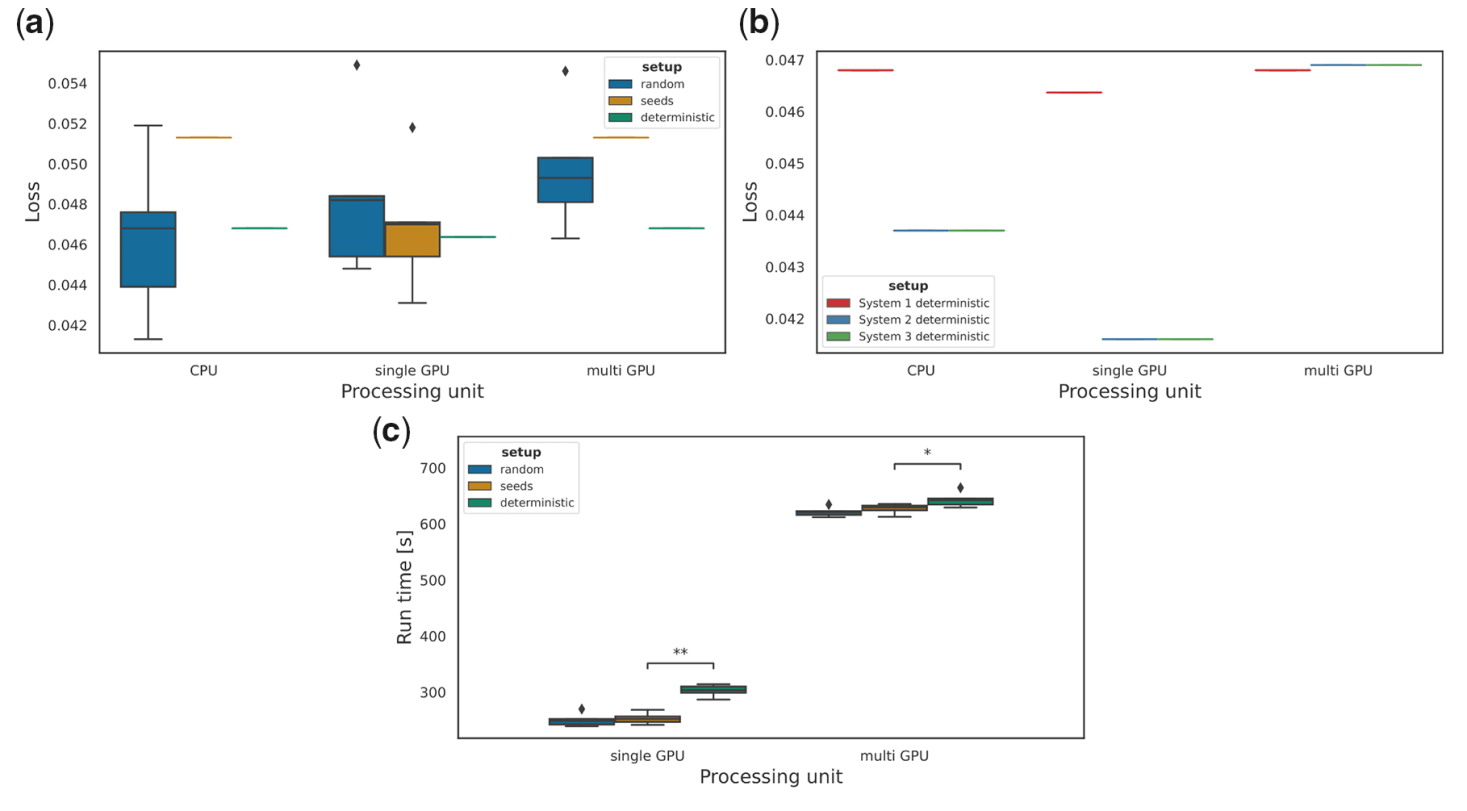
Determinism evaluation of a convolutional neural network with dropout layers implemented in TensorFlow 2.2 and trained on the MNIST dataset. (a) Loss variation across five training runs (*N* = 5) after 25 training epochs. Different settings were compared: without random seeds or deterministic algorithms (random), solely setting the same library random seeds across runs (seeds); or setting the random seeds and enabling the deterministic algorithms for TensorFlow as implemented in the mlf-core ecosystem (deterministic). Training runs on CPU, single GPUs and multi-GPU setup in the same system are compared. (b) Loss comparison of five runs (*N* = 5) across individual systems with different hardware (Systems 1 and 2/3), and individual systems with the same hardware (Systems 2 and 3), all with deterministic settings. (c) Training run time for 25 epochs when training the model without setting random seeds, when setting the random seeds and when forcing deterministic algorithms. For each setting, five individual runs were considered (*N* = 5)

To empirically validate that the mlf-core ecosystem ensures the implementation of deterministic models, we assessed the determinism of three ML models implemented with and without the determinism requirements employed by the mlf-core ecosystem. A two-layer convolutional neural network (CNN) model was trained to classify digits using the MNIST dataset, programmed with the PyTorch (Paszke et al. 2019) and TensorFlow (Abadi et al. 2015) libraries. Determinism for gradient-boosted trees implemented with XGBoost (Chen and Guestrin 2016), a distributed gradient-boosting library, was evaluated using the Covertype dataset. All evaluations were conducted on systems using a CPU, a single GPU, and multiple GPUs (Supplementary Table S1). Additionally, three different training setups were tested: (i) A setup without specified seeds or any deterministic setting (*random*), (ii) a setup that specified random seeds for all libraries used for the model implementation (*seeds*), and (iii) a setup which specified all random seeds, and additionally forced deterministic algorithms together with disabled cuDNN benchmark functionality, as implemented in the mlf-core ecosystem (*deterministic*) (Supplementary Fig. S3). Figure 3a shows the loss value of the last training iteration of the TensorFlow two-layer CNN implementation for five training repetitions. Only the deterministic setup implemented with mlf-core achieved fully deterministic results on all tested infrastructures, including a single CPU, a single GPU and a multi-GPU setup (Fig. 3a for the TensorFlow implementation, Supplementary Figs S4–S6 for the PyTorch and XGBoost implementations, respectively and Supplementary Fig. S6 for the weight standard deviations).

Even when all deterministic settings were enabled, the same models trained on systems with different hardware generated different results, emphasizing the need of hardware tracking when training models (Fig. 3b). Training the model in two machines with identical hardware resulted in the exact same loss as demonstrated with Systems 2 and 3 (Fig. 3b). These deterministic results could be reproduced for PyTorch (Supplementary Fig. S4). The evaluation of the XGBoost library unveiled nondeterminism when using XGBoost version 1.0.2 and XGBoost versions 1.1.0 compiled with CUDA 9 (Supplementary Fig. S5). However, training models with XGBoost version 1.1.0 compiled with CUDA 10 on a single GPU led to fully deterministic results. Regardless, runs with multiple GPUs on all systems with XGBoost did not result in deterministic runs, suggesting that XGBoost models should be trained on single GPUs to ensure determinism (Supplementary Fig. S5).

Next, we investigated the influence of forced determinism requirements on model training runtimes. Enabling *deterministic* algorithms resulted in significantly higher run times both when training on a single GPU (two-tailed t-test, *P* = .001) and multiple GPUs (two-tailed *t*-test, *P* = .014) when comparing the *seed* and *deterministic* settings for TensorFlow (Fig. 3c). Runs with PyTorch and deterministic algorithms enabled did not result in significantly higher runtimes compared to the *seeds-only* setting (two-tailed *t*-test *P* = .5558) (Supplementary Fig. S4) for a single GPU. For multiple GPUs, the runtime was significantly higher when *deterministic* settings were enabled (two-tailed t-test *P* = .001). These results indicate that deterministic models might result in longer runtimes.

### 3.3 mlf-core biomedical use cases

To showcase the applicability and advantages of the mlf-core ecosystem for deterministic and reproducible ML in various biomedical fields, we implemented three use cases, each one based on one of the three ML libraries supported by mlf-core.

#### 3.3.1 mlf-core enables reproducible analysis of scRNA-seq data with TensorFlow

**Figure 4:**
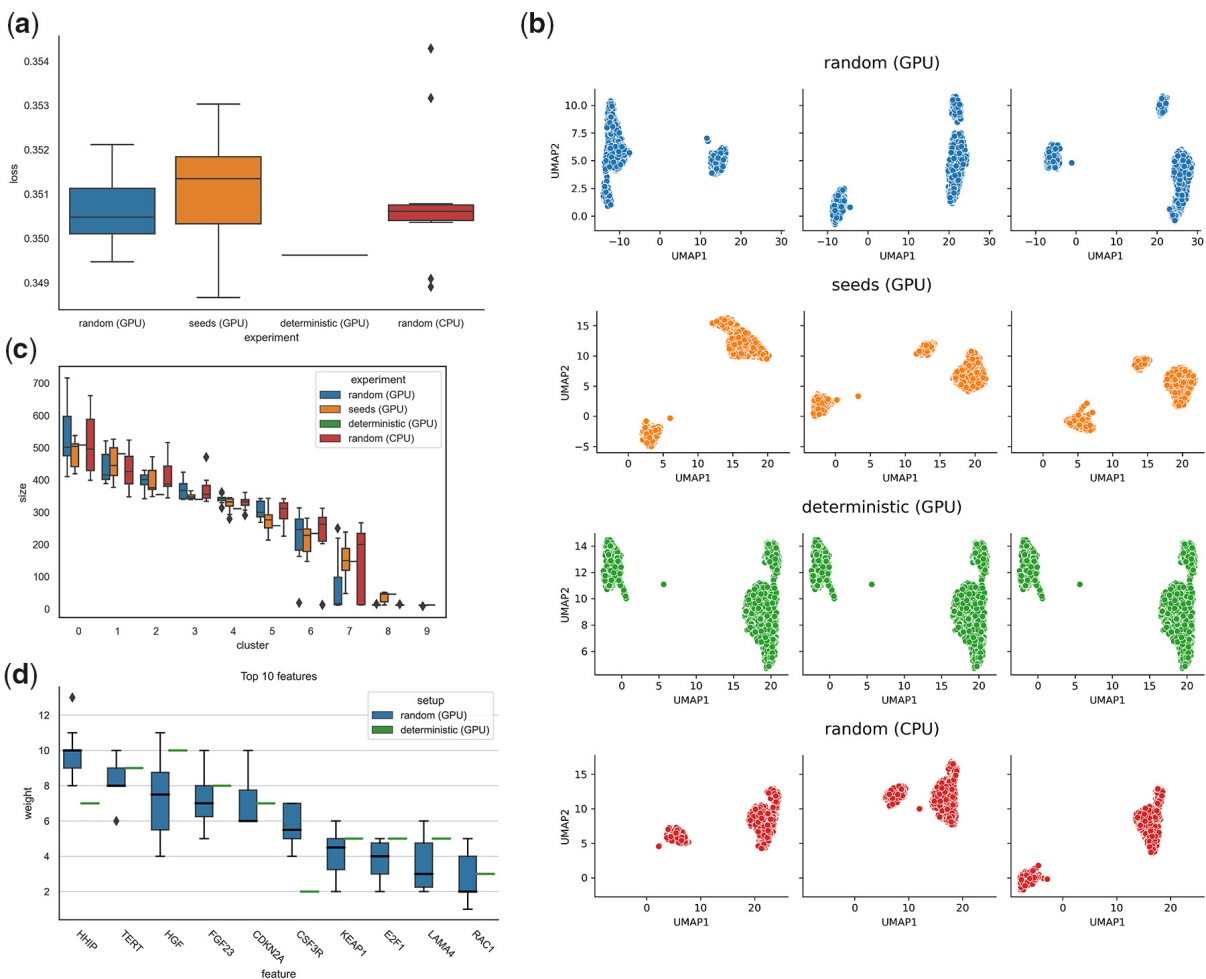
Impact of nondeterministic settings on an autoencoder model for single-cell RNA-seq data and on a XGboost classification model on a liver cancer dataset. (a) Loss variation of an autoencoder trained on single-cell RNA-seq data after 1000 epochs of training with no random seeds set (random), all possible random seeds set (seeds) and deterministic algorithms forced (deterministic). For each setting, 10 different runs were performed (*N* = 10). (b) UMAP plots generated from the autoencoder embedding after training for 1000 epochs in different experimental settings. Each row corresponds to a different setting and each column to a different run with the same settings. (c) Boxplots of cluster sizes for different experimental settings. Clusters were generated using the Leiden clustering algorithm and the autoencoder embedding. Different clusters are marked at the x-axis, boxplot colors correspond to experimental settings. (d) The 10 features with the highest average weight of the nondeterministic XGBoost model on liver cancer. For each setup, 10 runs were performed (*N* = 10). The features were ordered by decreasing average weight

Deep learning-based methods are commonly used for the analysis of scRNA-seq data, which is ideally suited for ML due to high sample numbers ranging from thousands to millions of cells. For instance, unsupervised methods such as autoencoders have been successfully applied in a variety of settings (Eraslan et al. 2019, Lotfollahi et al. 2019; Badsha et al. 2020; Chi and Deng 2020). Given the frequent use of deep learning models for scRNA-seq data, we wanted to evaluate how randomness affects the reproducibility of downstream analyses. To test this, we fitted a simple autoencoder model to a peripheral blood mononuclear cell scRNA-seq dataset. The comparison of the loss after and during training shows differences for nondeterministic experiments, even if no convolutional layers are used (Fig. 4a, Supplementary Fig S8a, b, and d).

We furthermore used the autoencoder latent space as input to Uniform Manifold Approximation and Projection (UMAP) (McInnes et al. 2018), a frequently used algorithm for visualizing scRNA-seq data in two dimensions. Nondeterministic models lead to visibly different UMAP plots, which hinder comparison and thus reproducibility (Fig. 4b). Finally, we performed clustering of cells based on the latent space embeddings, which is essential to most scRNA-seq data analyses. Comparison of cluster sizes for different runs showed considerable differences among the nondeterministic runs which would result in biologically relevant different cell type annotations (Fig. 4c). When applying the deterministic settings, as provided by the mlf-core framework, the autoencoder loss reached always the same value among the different runs (Fig. 4a). Moreover, the cluster sizes and UMAP projection remained identical (Fig. 4b and c).

#### 3.3.2 mlf-core enables deterministic feature importance determination with XGBoost

ML can be applied to identify novel oncology projects where data on large patient cohorts are available to identify novel biomarkers for diagnostics or potential targets (Way et al. 2018; van IJzendoorn et al. 2019). One widely used unsupervised approach to identify novel biomarkers is to train a model on gene expression data and to analyze the model feature importance assigned to the genes (Herman et al. 2018; Mamoshina et al. 2018). However, if no measures are taken to ensure reproducibility during the ML analysis, the importance assigned to the gene features will vary among different runs of the same model. To showcase this effect, we applied a feature importance determination approach to hepatocellular carcinoma (HCC), the most frequent type of liver cancer (El-Serag and Rudolph 2007). Using an RNA-Seq-derived gene expression dataset consisting of publicly accessible data, we trained a gradient boosting model (XGBoost) to classify gene expression profiles into malignant and healthy.

To evaluate the determinism of this model, we compared the loss values (log loss) among different runs with deterministic settings as provided by mlf-core, to the loss obtained from runs without deterministic settings. When determinism was not controlled, the model training loss values differed across the runs. The average weights of the 10 most important features of nondeterministic XGBoost models show considerable variances across the runs (Fig. 4d). The order of these features also changes in different runs with nondeterministic settings. This is illustrated in Supplementary Fig. S7, which shows the weights assigned to each of the features for each of the runs with nondeterministic settings. The exact values and feature names are also summarized in Supplementary Table S2. When setting the seeds to obtain deterministic results, the loss values and feature important weights stayed constant across all runs (Fig. 4d).

#### 3.3.3 mlf-core enables reproducible semantic segmentation of liver CT scans with PyTorch

CT is commonly used for liver-tumor evaluation and staging. It allows the study of anomalies in the shape and texture of liver tissue, which are important biomarkers for initial disease diagnosis and progression (Heimann et al. 2009). In this setting, volumetric segmentation of liver and malignant tissue in CT scans is an important task for cancer diagnosis and treatment, e.g. it allows the calculation of tumor burden (liver/tumor ratio) to assess disease progression and effectiveness of treatment (Blachier et al. 2013).

We evaluated the performance and reproducibility of a U-Net architecture (Ronneberger et al. 2015) (Supplementary Fig. S9) trained on the *Liver-Tumor Segmentation Benchmark* (LiTS) dataset (Bilic et al. 2019), since this model has been widely applied for semantic segmentation of microscopy and medical imaging data (Chlebus et al. 2018; Belthangady and Royer 2019; Jin et al. 2020; Moebel et al. 2020). The training dataset of LiTS comprises 131 abdominal CT scans of patients with HCC, with the corresponding ground-truth segmentation of liver and tumor lesions, as annotated by experts (Supplementary Fig. S10).

**Figure 5:**
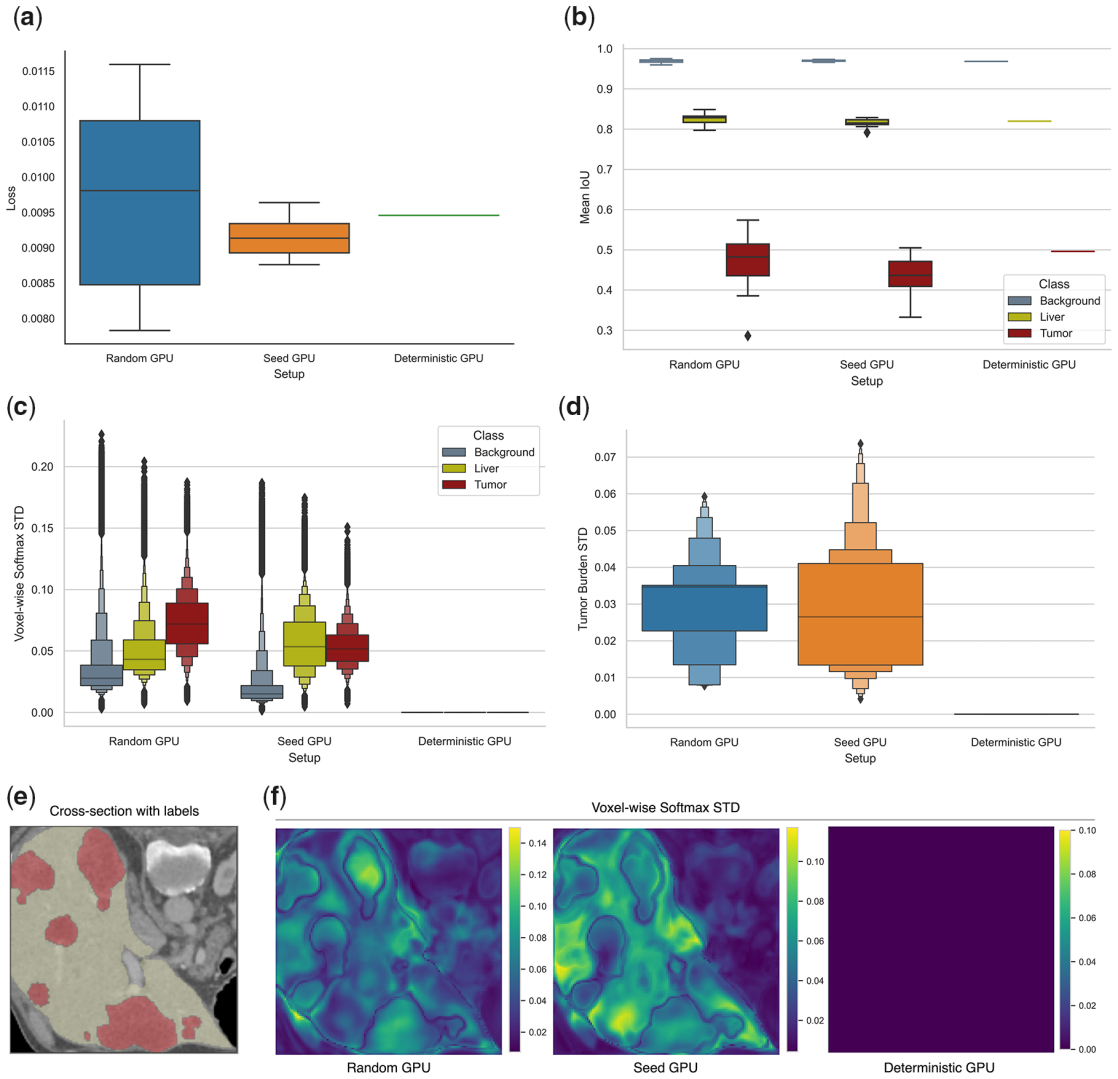
Impact of nondeterministic settings on a U-Net model for semantic segmentation trained on the LiTS dataset and implemented with PyTorch. (a) Boxplot of final losses after 1000 epochs of training with no random seeds set (random), all possible random seeds set (seeds) and deterministic algorithms forced (deterministic). For each setting, 10 different runs were performed (*N* = 10). (b) Performance of the trained models on the testing set (13 tomograms) for different experimental setups, showing mean IoU values per class (*N* = 10). (c) Letter-value (Hofmann et al. 2017) plot of standard deviations (STD) from the voxel-wise, softmax values used for prediction, as computed by trained models with different experimental setups (*N* = 10). (d) Letter-value plot of standard deviation values of tumor burden measurements from the testing set, using the segmentation masks predicted by the trained models (*N* = 10). (e) Cross-section of a tomogram from the test set with the ground-truth class labels, color coding voxels for the liver class and tumor class. (f) The same cross-section as (e), showing the standard deviation of the voxel-wise softmax values among 10 runs (*N* = 10), for the three experimental setups

We randomly sampled 10% of the dataset to define a small test set (13 tomograms) and trained our models for 1000 epochs with the remaining tomograms using different experimental GPU setups. We compared the final loss between runs and did not observe any variation of the deterministic setup, as opposed to the *random* and *seed* setups (Fig. 5a). We tested the reproducibility of prediction by evaluating the performance of the models on the abovementioned test set, using *Intersection over Union* (IoU) as a metric (Fig. 5b). Additionally, we calculated voxel-wise segmentation accuracy, and metrics for tumor lesion detection, specifically detection precision and F1-score (Supplementary Fig. S11). We observed consistent results across all performance metrics. Likewise, we observed no differences in IoU for the deterministic setup, while other setups exhibited noticeable variation, in particular for the tumor class. We calculated the standard deviation of the voxel-wise, softmax value used for prediction on the test set (Fig. 5c), and could verify that these values are reproducible using the deterministic setup. Moreover, we used the predicted segmentation masks to compute tumor burden for each tomogram in the test set, and measured the standard deviation across runs (Fig. 5d). We could only observe reproducible tumor burden calculations using the deterministic setup. We verified the same behavior on the epoch-wise loss and model parameters (Supplementary Fig. S12a and b).

## 4 Discussion

In the current work, we introduce a framework to ensure deterministic settings in ML projects. Setting random seeds for all the libraries during model training has proven necessary but not sufficient to ensure the bit-exact reproducibility of the model metrics and subsequently the performed predictions (Fig. 3, Supplementary Figs S4 and S5). This is due to the fact that ML libraries use nondeterministic algorithms based on atomic operations when training with GPUs, to ensure the fastest training. The large number of available cores of GPUs allows for massively parallel processing of simple tasks such as additions or multiplications which are employed during the training of (convolutional) neural networks. However, the employed atomic operations result in nondeterminism due to the nonassociativity of floating-point arithmetics (Chen and Feng 2013; Ahrens et al. 2020). Applied in large numbers, *atomic add* based sum reduce algorithms lead to a significant accumulation of floating-point errors. Functions such as bias additions and max-pooling, which are based on atomic add and batch normalization, will therefore produce nondeterministic results (Pham et al. 2020). When replacing the atomic add operations with deterministic equivalents the bias additions become deterministic, but potentially at a speed loss. ML frameworks such as PyTorch, TensorFlow, and XGBoost still allow for the usage of several less commonly used algorithms based on *atomic add* (https://pytorch.org/docs/stable/notes/randomness.html and https://github.com/NVIDIA/framework-determinism). So far, equivalent, deterministic algorithms are not available for these functions. Enforcing deterministic algorithms through machine library-specific flags is also not sufficient, because many common algorithms (e.g. 3D convolution) are still implemented nondeterministically based on atomic operations. The mlf-core linting tool integrated into the mlf-core framework warns developers when nondeterministic algorithms are being used.

Caution should be taken when using functionality such as *cuDNN benchmark*, a function from the NVIDIA cuDNN library, which selects the most appropriate algorithm taking as a criteria the fastest implementation. *cuDNN benchmark* can choose among deterministic and nondeterministic algorithms, making it difficult to evaluate determinism as it can be switched on and off in different experiments. The observed determinism of TensorFlow (Fig. 3a) when training with multiple GPUs may be due to *cuDNN benchmark* selecting deterministic variants for the used algorithms. However, it cannot be guaranteed that the same algorithms will be selected when training the models on different systems with the same hardware. Hence, deactivating *cuDNN benchmark* and enforcing deterministic algorithms is key to ensure the bit-exact same results. Since not all algorithms currently have deterministic equivalents, further work to implement deterministic versions of nondeterministic counterparts is imperative.

The selection of deterministic algorithms for all computations might come at the cost of a higher runtime necessary for training, as observed for the TensorFlow model trained on the MNIST dataset (Fig. 3c) (Pham et al. 2020). However, we did not observe increased runtimes while evaluating a similar model implemented in PyTorch, where the runtime was even lower with deterministic algorithms enabled (Supplementary Fig. S4d). Moreover, runtimes of the significantly larger 3D U-Net model, trained on the LiTS dataset, and implemented in PyTorch, did not increase but were shorter on average (Supplementary Fig. S10c). In fact, we argue that forcing deterministic algorithms even for extremely large-scale models will benefit development time in the long run since debugging, experimentation, and regression testing are greatly simplified.

Data distribution over multiple worker instances with Dask (Rocklin 2015), a cluster often employed to distribute over several GPUs, is not deterministic (Supplementary Fig. S4). When training a model on multiple GPUs on a Dask cluster every GPU is treated as a standalone worker. Dask requires the data to be distributed and partitioned across workers, which for maximum speed currently does not guarantee determinism. Therefore, the usage of multiple worker instances either in the form of CPUs or GPUs is not recommended when deterministic ML is desired.

The software stack has to stay consistent to ensure determinism since different CUDA drivers or ML algorithm implementations affect the generated results (Supplementary Fig. S4). A further issue usually hidden from the end user is that the distributed packages have to be compiled with the correct dependency versions. The hardware stack also plays a major role in determinism. Different GPU architectures partition the computational load for massively parallel processing in distinct ways. Any changes in this partitioning will be reflected in differences in floating-point rounding error accumulation (Demmel et al. 2016; Nagarajan et al. 2019) (Fig. 3). Cloud computing greatly simplifies access to standardized hardware which together with mlf-core’s logging of used hardware mitigate this issue allowing for portable deterministic ML.

We summarize the technical requirements to ensure deterministic results based on the aforementioned causes for nondeterminism identified in this work as the following points:

- **Setting random seeds:** All random seeds used by all imported libraries need to be set.
- **Using exclusively deterministic algorithms:** The use of deterministic algorithms must be enforced and ensured that nondeterministic algorithms cannot be selected. Furthermore, functionality such as *cuDNN Benchmark*, which chooses algorithms at runtime based on their performance in different hardware, must be switched off.
- **Containerizing the runtime environment:** The complete runtime environment must be containerized to ensure a consistent runtime environment and control the versions of all software used for model training.
- **Logging the hardware:** The used hardware architecture must be documented, especially the used CPU and all GPUs, and kept constant for reproducibility.
- **Documenting model training and parameters, and sharing the code:** All hyperparameters, obtained metrics, and model details must be documented together with the full open-source code for all model training runs.

The mlf-core framework enforces all the aforementioned requirements for deterministic ML directly into the model code and thus provides the first complete solution for CPU and GPU reproducible ML (Supplementary Fig. S13). The ML templates provided by mlf-core automatically set all the necessary random seeds for the Tensorflow, PyTorch, or XGBoost libraries. The mlf-core linting functionality ensures that nondeterministic functions are not employed and warns the user if selected functions are nondeterministic. mlf-core supports Docker to ensure a consistent runtime environment, as well as tracking of the hardware employed for ML training with the system intelligence tool. The seamless integration with MLFlow ensures the documentation of hyperparameters, training metrics and model details for each of the training runs. The very high degree of automation and ease of use opens the framework to a wide audience.

We showcased the usage of the mlf-core ecosystem to develop deterministic ML models for three different use cases relevant for biomedical data science. Using TensorFlow and simple autoencoder models to process scRNA-seq, our data showed that nondeterministic algorithms have an impact on the reproducibility of scRNA-seq data analysis, leading to differences in cell clustering and thus potentially cell-type assignments.

The liver cancer classifier based on the mlf-core XGBoost template highlights the importance of deterministic algorithms and set seeds when extracting feature importance. Life scientists base further experiments on candidate gene importance and therefore need reproducible computational models especially when new data is regularly added to the experiment.

For semantic segmentation of CT scans using PyTorch, we showed that the deterministic setup is able to produce reproducible results using a simplified U-Net model, both during training and for prediction. Enforcing determinism of models used for liver-tumor segmentation of abdominal CT scans is critical to ensure reproducibility in patient diagnostics, especially when measuring tumor burden or applying the *Response Evaluation Criteria in Solid Tumor* (RECIST) protocol for tumor staging (Eisenhauer et al. 2009).

In practice, we suggest that researchers developing new models should try to evaluate and reduce the variance of the model’s training and performance metrics as much as possible through architectural adaptations, hyperparameter selections, and model initializations, with deterministic algorithms disabled. In a second step, deterministic algorithms should be forced using mlf-core to select the deterministic model from the pool of low variance models. This ensures that the deterministic model is as representative as possible.

Our work unveils the causes of nondeterministic ML and we provide the first solution to enable deterministic predictions: the mlf-core framework. We envision mlf-core to enable the ML community to develop deterministic ML models, advancing research while increasing society’s trust in deployed models.

## Supplementary Material

btad164_Supplementary_Data

## Data Availability

Code and links to data resources used to build this manuscript and its figures, can be found in the paper’s public repository: https://github.com/mlf-core.

## References

[btad164-B1] Abadi M , Agarwal A, Barham P et al *TensorFlow: Large-Scale Machine Learning on Heterogeneous Systems.* arXiv 2015;1603.04467.

[btad164-B2] Ahrens P , Demmel J, Nguyen HD. Algorithms for efficient reproducible floating point summation. ACM Trans Math Softw 2020;46:1–49.

[btad164-B3] *Anaconda Software Distribution.* Computer Software. Vers. 2-2.4.0. 2016.

[btad164-B4] Badsha MB , Li R, Liu B et al Imputation of single-cell gene expression with an autoencoder neural network. Quant Biol (Beijing, China) 2020;8. 10.1007/s40484-019-0192-7 PMC7144625 32274259

[btad164-B5] Beam AL , Kohane IS. Big data and machine learning in health care. JAMA 2018;319:1317. 29532063 10.1001/jama.2017.18391

[btad164-B6] Belthangady C , Royer LA. Applications, promises, and pitfalls of deep learning for fluorescence image reconstruction. Nat Methods 2019;16:1215–25. 31285623 10.1038/s41592-019-0458-z

[btad164-B7] Bilic P , Christ PF, Vorontsov E et al The liver tumor segmentation benchmark (LiTS). Medical Image Analysis 2023;84:102680. 10.1016/j.media.2022.102680 PMC10631490 36481607

[btad164-B8] Blachier M , Leleu H, Peck-Radosavljevic M et al The burden of liver disease in Europe: a review of available epidemiological data. J Hepatol 2013;58:593–608. 23419824 10.1016/j.jhep.2012.12.005

[btad164-B9] Chen T , Feng B. Research on error accumulative sum of single precision floating point. J Comput Appl 2013;33:1531–3.

[btad164-B10] Chen T , Guestrin C. XGBoost: a scalable tree boosting system. In: *Proceedings of the 22nd ACM SIGKDD International Conference on Knowledge Discovery and Data Mining, San Francisco.* New York, NY, United States: Association for computing machinery. 785–94. 2016.

[btad164-B11] Chi W , Deng M. Sparsity-Penalized stacked denoising autoencoders for imputing single-cell RNA-Seq data. Genes 2020;11:532. 32403260 10.3390/genes11050532 PMC7291078

[btad164-B12] Chlebus G , Schenk A, Moltz JH et al Automatic liver tumor segmentation in CT with fully convolutional neural networks and object-based postprocessing. Sci Rep 2018;8:15497. 30341319 10.1038/s41598-018-33860-7 PMC6195599

[btad164-B13] Çiçek Ö , Abdulkadir A, Lienkamp SS et al 3D U-Net: learning dense volumetric segmentation from sparse annotation. In: *Medical Image Computing and Computer-Assisted Intervention – MICCAI 2016*. Lecture Notes in Computer Science, Vol. 9901. Springer 424–32. 2016.

[btad164-B14] Collberg C , Proebsting TA. Repeatability in computer systems research. Commun ACM 2016;59:62–9.

[btad164-B15] Demmel J , Ahrens P, Nguyen HD. Efficient Reproducible Floating Point Summation and BLAS. Berkeley: EECS Department, University of California, 2016.

[btad164-B16] Eisenhauer EA , Therasse P, Bogaerts J et al New response evaluation criteria in solid tumours: revised RECIST guideline (version 1.1). Eur J Cancer 2009;45:228–47. 19097774 10.1016/j.ejca.2008.10.026

[btad164-B17] El-Serag HB , Rudolph KL. Hepatocellular carcinoma: epidemiology and molecular carcinogenesis. Gastroenterology 2007;132:2557–76. 17570226 10.1053/j.gastro.2007.04.061

[btad164-B18] Eraslan G , Simon LM, Mircea M et al Single-cell RNA-seq denoising using a deep count autoencoder. Nat Commun 2019;10:1–14. 30674886 10.1038/s41467-018-07931-2 PMC6344535

[btad164-B19] Ewels PA , Peltzer A, Fillinger S et al The nf-core framework for community-curated bioinformatics pipelines. Nat Biotechnol 2020;38:276–8. 32055031 10.1038/s41587-020-0439-x

[btad164-B20] Haibe-Kains B , Adam GA, Hosny A, et al; Massive Analysis Quality Control (MAQC) Society Board of Directors. Transparency and reproducibility in artificial intelligence. Nature 2020;586:E14–6. 33057217 10.1038/s41586-020-2766-y PMC8144864

[btad164-B21] Heil BJ , Hoffman MM, Markowetz F et al Reproducibility standards for machine learning in the life sciences. Nat Methods 2021;18:1132–5. 34462593 10.1038/s41592-021-01256-7 PMC9131851

[btad164-B22] Heimann T , van Ginneken B, Styner MA et al Comparison and evaluation of methods for liver segmentation from CT datasets. IEEE Trans Med Imaging 2009;28:1251–65. 19211338 10.1109/TMI.2009.2013851

[btad164-B23] Henderson P , Islam R, Bachman P et al Deep reinforcement learning that matters. In: *Proceedings of the Thirty-Second AAAI Conference on Artificial Intelligence and Thirtieth Innovative Applications of Artificial Intelligence Conference and Eighth AAAI Symposium on Educational Advances in Artificial Intelligence (AAAI'18/IAAI'18/EAAI'18)*. AAAI Press, Article 392, 3207–3214.

[btad164-B24] Herman JS , Grün D, Sagar. FateID infers cell fate bias in multipotent progenitors from single-cell RNA-seq data. Nat Methods 2018;15:379–86. 29630061 10.1038/nmeth.4662

[btad164-B25] Heumos L , Ehmele P, Langes T et al cookiejar/cookietemple: [1.2.2 Release]. Zenodo. 2020.

[btad164-B26] Hofmann H , Wickham H, Kafadar K. Letter-Value plots: boxplots for large data. J Comput Graph Stat 2017;26:469–77.

[btad164-B27] Hutson M. Artificial intelligence faces reproducibility crisis. Science 2018;359:725–6. 29449469 10.1126/science.359.6377.725

[btad164-B28] Jin Q , Meng Z, Sun C et al RA-UNet: a hybrid deep attention-aware network to extract liver and tumor in CT scans. Front Bioeng Biotechnol 2020;8:605132. 33425871 10.3389/fbioe.2020.605132 PMC7785874

[btad164-B29] Kruppa J , Schwarz A, Arminger G et al Consumer credit risk: individual probability estimates using machine learning. Expert Syst Appl 2013;40:5125–31.

[btad164-B30] Lotfollahi M , Wolf A, Theis F et al scGen predicts single-cell perturbation responses. Nat Methods 2019;16:715–21. 31363220 10.1038/s41592-019-0494-8

[btad164-B31] Mamoshina P , Volosnikova M, Ozerov IV et al Machine learning on human muscle transcriptomic data for biomarker discovery and tissue-specific drug target identification. Front Genet 2018;9:242. 30050560 10.3389/fgene.2018.00242 PMC6052089

[btad164-B32] Matschinske J , Alcaraz N, Benis A et al The AIMe registry for artificial intelligence in biomedical research. Nat Methods 2021;18:1128–31. 34433960 10.1038/s41592-021-01241-0

[btad164-B33] McInnes L , Healy J, Melville J. *UMAP: Uniform Manifold Approximation and Projection for Dimension Reduction.* arXiv 2018;1802.03426.

[btad164-B34] Merkel D. Docker: lightweight linux containers for consistent development and deployment. Linux J 2014;2014.

[btad164-B35] Moebel E , Martinez-Sanchez A, Lamm L et al Deep learning improves macromolecule identification in 3D cellular cryo-electron tomograms. Nat Methods 2021;18:1386–94. 10.1038/s41592-021-01275-4. 34675434

[btad164-B36] Mongan J , Moy L, Kahn CE Jr. Checklist for artificial intelligence in medical imaging (CLAIM): a guide for authors and reviewers. Radiol Artif Intell 2020;2:e200029. 33937821 10.1148/ryai.2020200029 PMC8017414

[btad164-B37] Nagarajan P , Warnell G, Stone P. Deterministic implementations for reproducibility in deep reinforcement learning. In: *2nd Reproducibility in Machine Learning Workshop at ICML 2018*, *Stockholm, Sweden, July 2018*.

[btad164-B38] Norgeot B , Quer G, Beaulieu-Jones BK et al Minimum information about clinical artificial intelligence modeling: the MI-CLAIM checklist. Nat Med 2020;26:1320–4. 32908275 10.1038/s41591-020-1041-y PMC7538196

[btad164-B39] Gundersen OE , Kjensmo S. State of the art: reproducibility in artificial intelligence. In: *Proceedings of the AAAI Conference on Artificial Intelligence*, *32*(1), Association for the Advancement of Artificial Intelligence.

[btad164-B40] Olorisade BK , Brereton P, Andras P. Reproducibility of studies on text mining for citation screening in systematic reviews: evaluation and checklist. J Biomed Inform 2017;73:1–13. 28711679 10.1016/j.jbi.2017.07.010

[btad164-B41] Paszke A , Gross S, Massa F et al PyTorch: an imperative style, high-performance deep learning library. In: H. Wallach, H. Larochelle, A. Beygelzimer, et al (eds.), Advances in Neural Information Processing Systems. Vol. 32. Red Hook, NY, USA: Curran Associates, Inc. 2019, 8024–35.

[btad164-B42] Pham HV , Qian S, Wang J et al Problems and opportunities in training deep learning software systems. In: *Proceedings of the 35th IEEE/ACM International Conference on Automated Software Engineering (ASE '20)*. New York, NY, USA: Association for Computing Machinery.

[btad164-B43] Rocklin M. Dask: parallel computation with blocked algorithms and task scheduling. In: *Proceedings of the 14th Python in Science Conference*. Python in Science Conference, Austin, Texas. 2015 SciPy Organizers.

[btad164-B44] Ronneberger O , Fischer P, Brox T. U-Net: convolutional networks for biomedical image segmentation. In: Navab, N., Hornegger, J., Wells, W., Frangi, A. (eds) *Medical Image Computing and Computer-Assisted Intervention – MICCAI 2015. Lecture Notes in Computer Science*, Vol. 9351. Springer:234–241.

[btad164-B45] Tayal DK , Jain A, Arora S et al Crime detection and criminal identification in India using data mining techniques. AI Soc 2015;30:117–27.

[btad164-B46] Toreini E , Aitken M, Coopamootoo KPL et al Technologies for trustworthy machine learning: a survey in a socio-technical context. arXiv 2020;2007.08911

[btad164-B47] van IJzendoorn DGP , Szuhai K, Briaire-de Bruijn IH et al Machine learning analysis of gene expression data reveals novel diagnostic and prognostic biomarkers and identifies therapeutic targets for soft tissue sarcomas. PLoS Comput Biol 2019;15:e1006826. 30785874 10.1371/journal.pcbi.1006826 PMC6398862

[btad164-B48] Way GP , Sanchez-Vega F, La K, et al; Cancer Genome Atlas Research Network. Machine learning detects pan-cancer RAS pathway activation in the cancer genome atlas. Cell Rep 2018;23:172–80.e3. 29617658 10.1016/j.celrep.2018.03.046 PMC5918694

[btad164-B49] Wilkinson MD , Dumontier M, Aalbersberg IJJ et al The FAIR guiding principles for scientific data management and stewardship. Sci Data 2016;3:160018. 26978244 10.1038/sdata.2016.18 PMC4792175

[btad164-B50] Zaharia M , Xin RS, Wendell P et al Apache spark: a unified engine for big data processing. Commun ACM 2016;59:56–65.

