# Supplementary material for: mlf-core: a framework for deterministic machine learning
Source: Bioinformatics. 2023 Apr 2;39(4):btad164. doi: 10.1093/bioinformatics/btad164 (PMC10089676; doi:10.1093/bioinformatics/btad164)
Supplement: btad164_Supplementary_Data [file btad164_supplementary_data.pdf]

mlf-core: a framework for deterministic machine learning

Lukas Heumos[1,2,3]*, Philipp Ehmele[4], Luis Kuhn Cuellar[1], Kevin Menden[1], Edmund Miller[5], Steffen Lemke[1], Gisela Gabernet[1#§], Sven Nahnsen[1,6,7,8#§]

[1] Quantitative Biology Center (QBiC) Tübingen, University of Tübingen, Tübingen, Germany
[2] Institute of Computational Biology, Helmholtz Zentrum München, Munich, Germany
[3] Institute of Lung Biology and Disease and Comprehensive Pneumology Center, Helmholtz Zentrum München, Munich, Germany
[4] Department of Informatics, University of Hamburg, Hamburg
[5] Department of Biological Sciences and Center for Systems Biology, The University of Texas at Dallas, Richardson, Texas, USA.
[6] Biomedical Data Science, Department for Computer Science, University of Tübingen, Tübingen, Germany
[7]Institute of Bioinformatics and Medical Informatics, Eberhard Karls University of Tübingen, Tübingen, Germany.
[8]Faculty of Medicine, Eberhard Karls University of Tübingen, Tübingen, Germany.
§ These authors contributed equally and share the senior authorship
** Corresponding author. E-mail: lukas.heumos@helmholtz-muenchen.de*
# Corresponding author: E-mail: gisela.gabernet@qbic.uni-tuebingen.de
# Corresponding author: E-mail: sven.nahnsen@qbic.uni-tuebingen.de

---

Supplementary information

Supplementary information 1
Supplementary tables 2
Supplementary figures 4
Supplementary Methods 17
PyTorch determinism evaluation setup 17
TensorFlow determinism evaluation setup 18
XGBoost determinism evaluation setup 18
Evaluating machine learning library determinism 19
Analysis of scRNA-seq data with TensorFlow 19
Use case XGBoost - Liver cancer data model 20
Liver tumor semantic segmentation with PyTorch 21
References 23

Supplementary tables

Supplementary Table 1. Overview about used hardware systems for determinism and runtime evaluations.

System	Hardware
1 - deNBI k80	Intel 12 core and 2 NVIDIA Tesla K80s
2/3 - denBI V100	Intel 24 core and 2 NVIDIA V100s

Supplementary Table 2. Feature weights of non-deterministic runs of the XGBoost liver cancer use case. The top 20 features are shown, sorted by decreasing mean weights.

Genes	run 1	run 2	run 3	run 4	run 5	run 6	run 7	run 8	run 9	run 10
HHIP	10	9	8	8	10	11	10	13	10	9
TERT	9	8	6	9	8	8	8	9	10	8
HGF	9	9	8	8	11	7	7	4	5	5
FGF23	6	8	10	8	6	8	7	7	7	5
CDKN2A	6	10	6	6	6	9	8	6	6	7
CSF3R	5	5	6	7	4	5	4	7	7	7
KEAP1	6	5	6	5	2	3	4	3	5	4
E2F1	3	4	4	4	5	5	3	3	5	2
LAMA4	5	6	3	3	2	2	5	4	3	2
RAC1	2	5	5	2	1	2	2	2	4	4
NOTCH3	1	3	3	4	4	3	3	2	1	3
EPO	5	3	1	1	3	1	2	3	2	3
CALML3	3	3	2	1	1	2	2	4	3	1
IL4R	2	1	1	2	3	4	4	1	2	2
ADCY1	3	2	1	3	3	0	1	3	3	3
DLL4	3	3	1	4	2	1	2	0	2	4
PDGFB	2	1	2	2	2	3	2	1	3	3
MET	2	4	2	2	2	2	4	0	2	0
EPOR	4	2	2	1	2	1	2	1	3	1
NRAS	3	2	1	3	2	1	0	3	3	1
PTK2	2	3	2	2	0	1	2	2	2	1
MAPK3	0	2	1	2	2	3	3	2	0	1
TCEB2	2	1	3	1	3	2	1	1	1	1
RHOA	4	1	0	0	3	3	2	3	0	0
GSTA5	1	1	1	2	3	0	1	2	3	1
CKS2	2	2	1	0	0	2	2	0	3	3
PIK3R3	0	0	3	2	0	1	0	3	4	1
NTRK1	1	2	0	2	2	2	0	1	2	1
F2RL3	0	1	2	1	1	2	1	4	1	0
FN1	0	2	0	1	1	3	0	3	0	2

Supplementary figures

a

```
1 import numpy as np
2 import torch
3 import os
4 import random
5
6 os.environ['PYTHONHASHSEED'] = SEED
7 random.seed(SEED)
8 np.random.seed(SEED)
9 torch.manual_seed(SEED)
10 torch.backends.cudnn.deterministic = True
11 torch.backends.cudnn.benchmark = False
12 # torch.set_deterministic(True)
```

b

```
1 import numpy as np
2 import tensorflow as tf
3 import os
4 import random
5
6 os.environ['PYTHONHASHSEED'] = SEED
7 random.seed(SEED)
8 np.random.seed(SEED)
9 tf.random.set_seed(SEED)
10 os.environ['TF_DETERMINISTIC_OPS'] = '1'
11 session_config.intra_op_parallelism_threads = 1
12 session_config.inter_op_parallelism_threads = 1
```

c

```
1 import numpy as np
2 import os
3 import random
4
5 os.environ['PYTHONHASHSEED'] = SEED
6 random.seed(SEED)
7 np.random.seed(SEED)
8 param = {'seed': SEED,
9          'single_precision_histogram': True}
```

Supplementary Figure 1. Set seeds and settings for the PyTorch, TensorFlow and XGBoost determinism evaluation. (a) All explicitly set seeds and PyTorch specific settings. *torch.backends.cudnn.deterministic* = *True* forces deterministic algorithms whenever available and *torch.backends.cudnn.benchmark* = *False* disables cuDNN benchmark which is cuDNN's inbuilt auto-tuner to find the fastest algorithms for the used hardware. Line 12 was not used for the evaluation since it was not available yet, but is used for the most recent mlf-core PyTorch project template. (b) All explicitly set seeds and TensorFlow specific settings. *torch.backends.cudnn.deterministic* = *True* forces deterministic algorithms when available and *torch.backends.cudnn.benchmark* = *False* disables cuDNN's inbuilt auto-tuner to find the fastest algorithms for the used hardware. (c) All explicitly set seeds and XGBoost specific settings. single_precision_histogram enables single precision histogram building to speed up training.

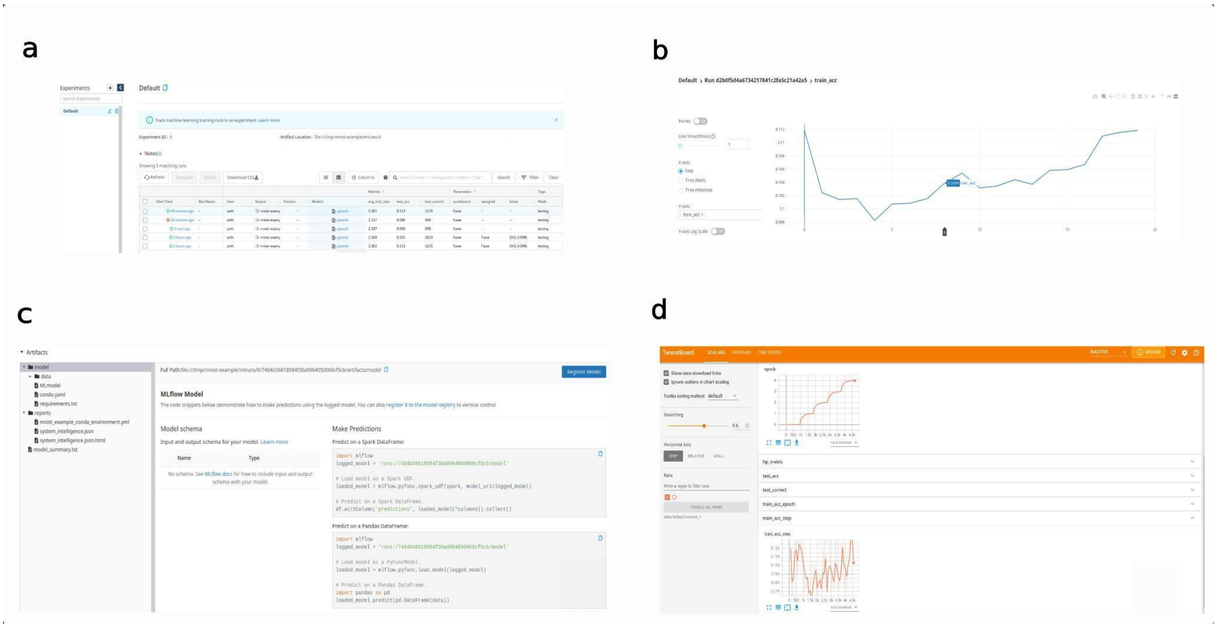

Supplementary Figure 2. Automatically generated interactive visualizations of the model training process with MLflow (a-c) and Tensorboard (d). (a) Overview of all currently running, failed and successfully completed model training runs associated with all hyperparameters and metrics. (b) Line plot of the training accuracy of an example training run per epoch. (c) Model artifacts of an example run including the pickled model and the system-intelligence hardware report. (d) Training accuracy per step of an example training run.

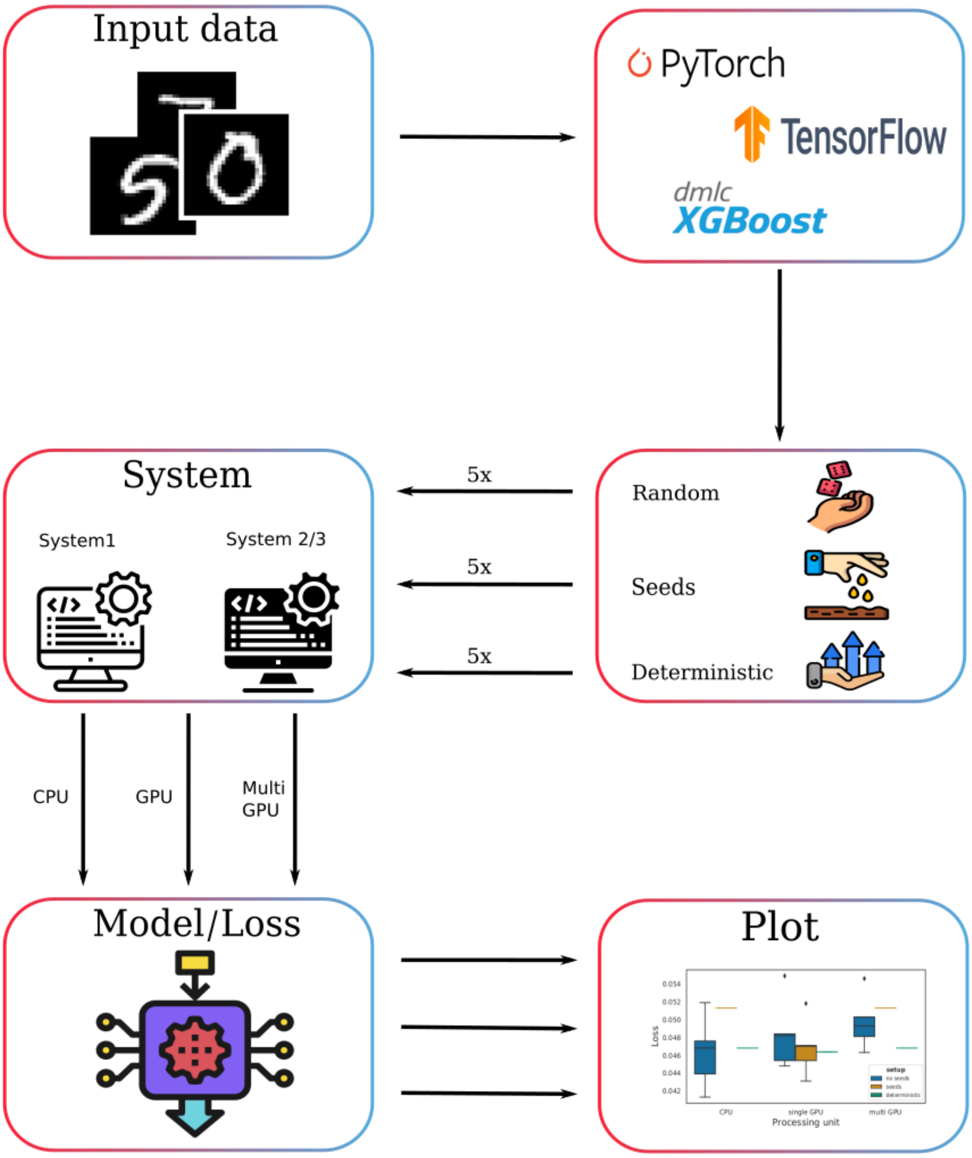

Supplementary Figure 3. Experimental setup for the determinism evaluation. PyTorch, TensorFlow and XGBoost models were trained with 3 setups. A random setup which did not specify any seeds, a seeds setup which set all seeds for the respective machine learning library and any underlying libraries and a deterministic which additionally forced deterministic algorithms and disabled cuDNN benchmark. All setups were trained 5 times for 3 hardware systems. System 2 and 3 shared the same CPU and GPU architectures. This setup was repeated for the CPU, a single GPU and multiple GPUs leading to several models and the corresponding losses.

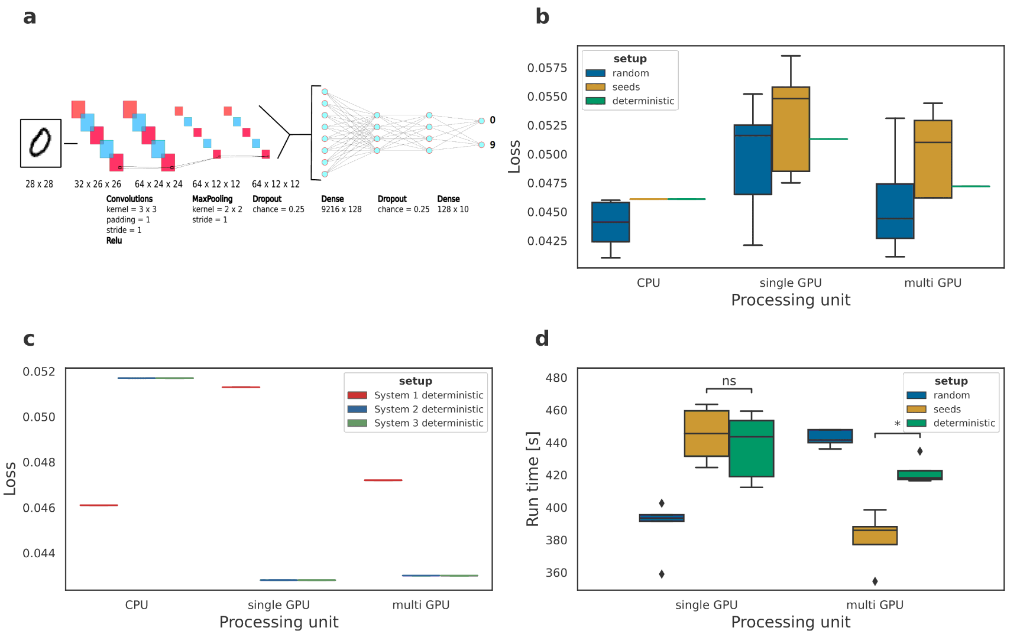

Supplementary Figure 4. Determinism evaluation of a simple convolutional neural network with dropout layers implemented in PyTorch 1.5 and trained on the MNIST dataset. (a) Neural network architecture for the PyTorch and TensorFlow determinism evaluation. (b) Loss variation across 5 model training runs in the same system with no random seeds or deterministic algorithms (random), solely setting the same library random seeds across runs (seeds); or setting the random seeds and enabling the deterministic algorithms (deterministic). (c) Loss comparison of 5 runs across individual systems with different hardware (systems 1 and 2/3), and individual systems with the same hardware (systems 2 and 3) with deterministic settings. (d) Training time for 25 epochs when training the model without setting random seeds, when setting the random seeds and when enabling the deterministic algorithms.

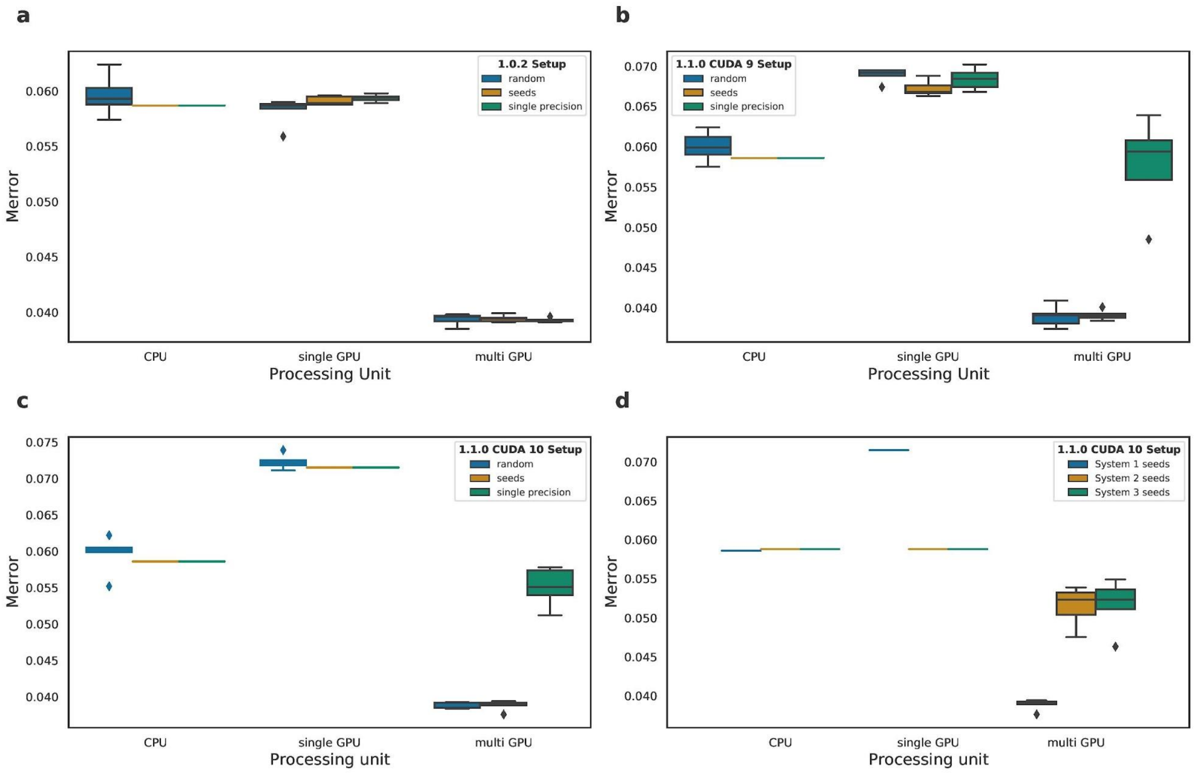

Supplementary Figure 5. Determinism evaluation of a simple Gradient Boosted Tree implemented in different XGBoost versions and trained on the Covertype dataset. (a) Multiclass classification error rate (Merror) variation across 5 model training runs (N=5) in the same system with no random seeds or deterministic algorithms (random); solely setting the same library random seeds across runs (seeds); or setting the random seeds and enabling single precision for XGBoost with XGBoost version 1.0.2 (single-precision). (b) XGBoost version 1.1.0 compiled with CUDA 9 and (c) XGBoost 1.1.0 compiled with CUDA 10. (d) Merror comparison of 5 runs across individual systems with different hardware (system 1 and 2/3) and individual systems with the same hardware (systems 2 and 3) with all required random seeds set.

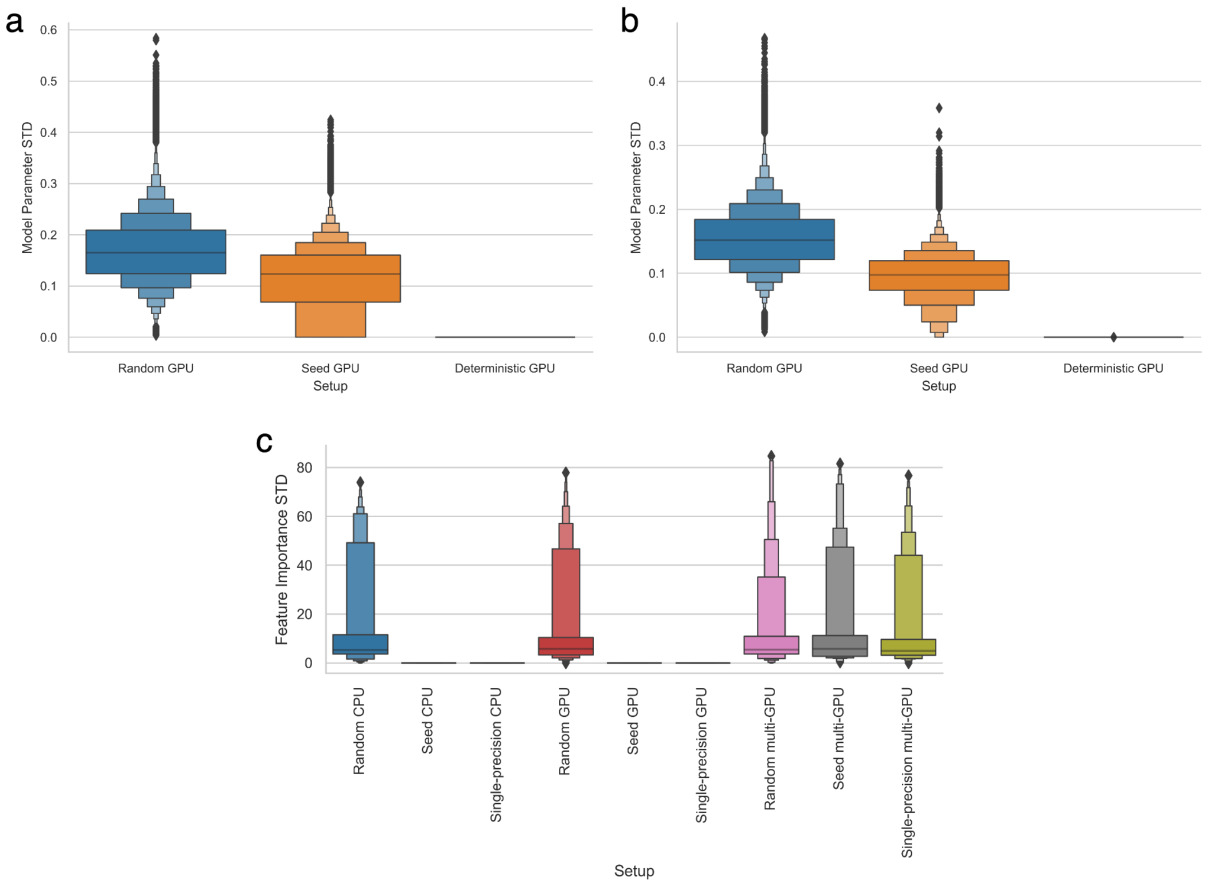

Supplementary Figure 6. Deviation of model parameters (weights and biases) and feature importance values after training runs, for the MNIST (PyTorch, TensorFlow) and CovType (XGBoost) datasets, in different experimental settings (setup). Models were trained 10 times on each setup (N=10). (a) Letter-value plot of standard deviations values of model parameters, for PyTorch models trained (100 epochs) on the MNIST dataset. (b) Letter-value plot of standard deviations values of model parameters, for Tensorflow models trained (100 epochs) on the MNIST dataset. (c) Letter-value plot of standard deviations of feature importance values from XGBoost models, trained on the CovType dataset.

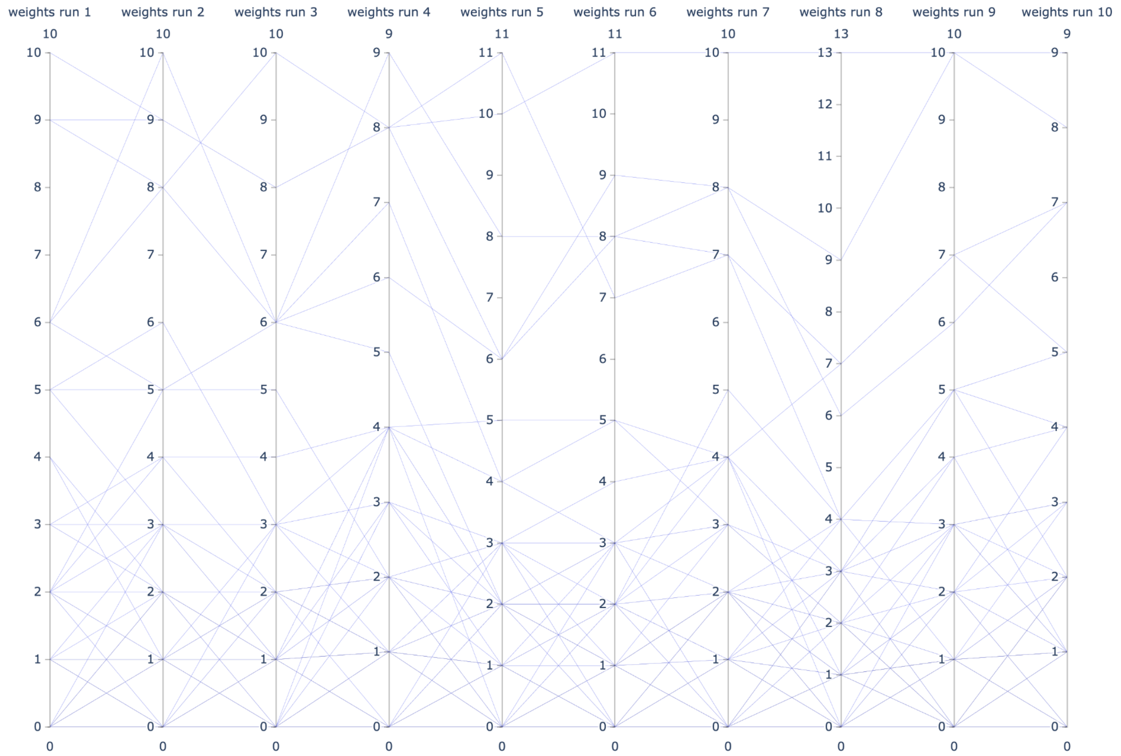

Supplementary Figure 7. Feature weights of ten non-deterministic runs of the XGBoost liver cancer use case. Each line represents one feature.

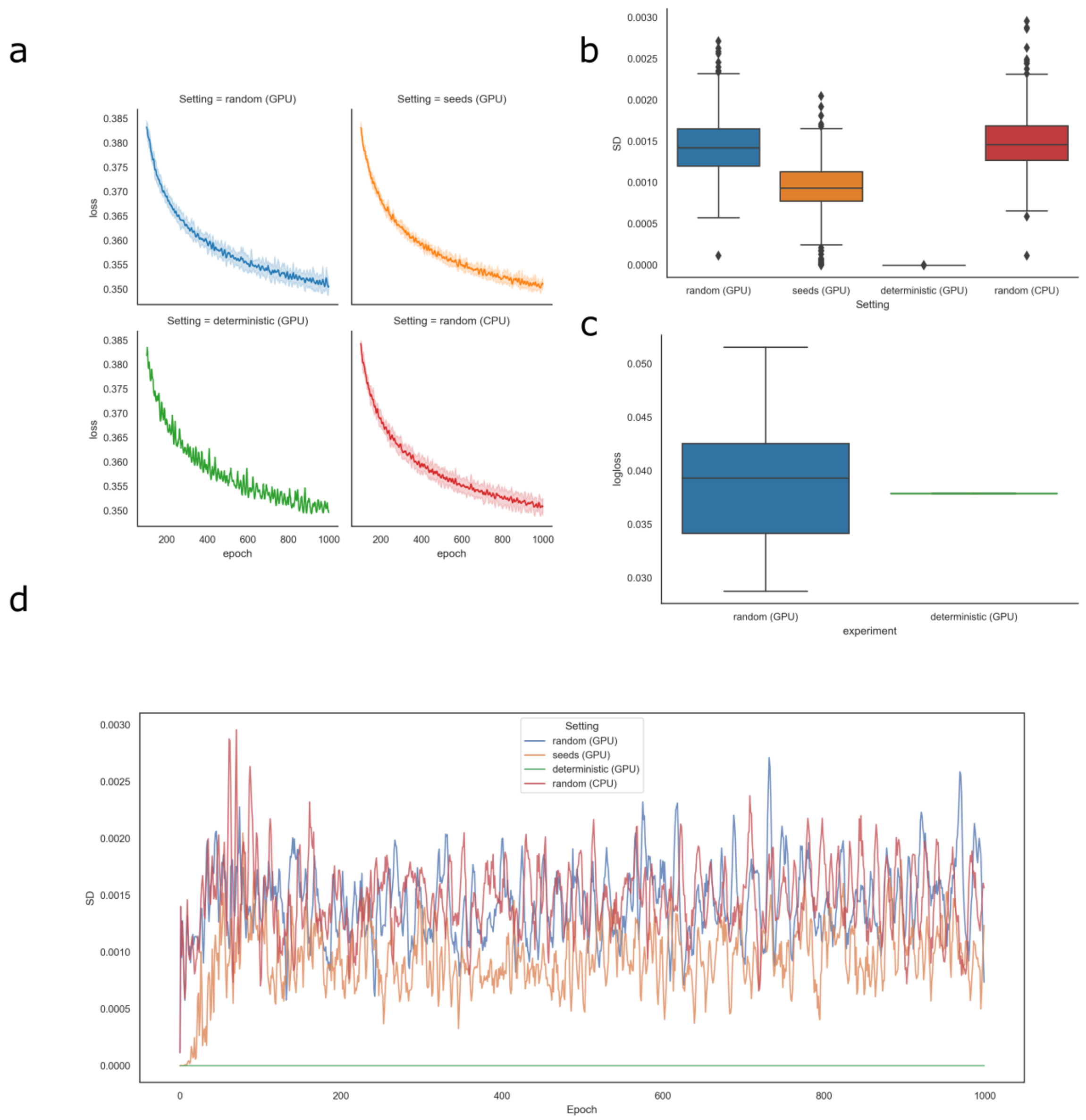

Supplementary Figure 8. Differences in standard deviations between losses of ten different runs (N=10) in different experimental settings for the single-cell autoencoder and liver cancer feature importance use cases. (a) Epoch-wise losses are shown for different experimental settings during training (starting from epoch 100) of the single-cell autoencoder. The standard deviation (SD) between separate runs in one setting are indicated with error bands. (b) Boxplots of the SDs of losses at every epoch, for every run and experimental setting of the single-cell autoencoder. (c) Loss (logloss) after 1000 epochs of the XGBoost liver cancer classifying model. For each setup, ten runs were performed. (d) SD per epoch plotted for every experimental setting and epoch of the single-cell autoencoder. The flat green line at the bottom represents the deterministic setting.

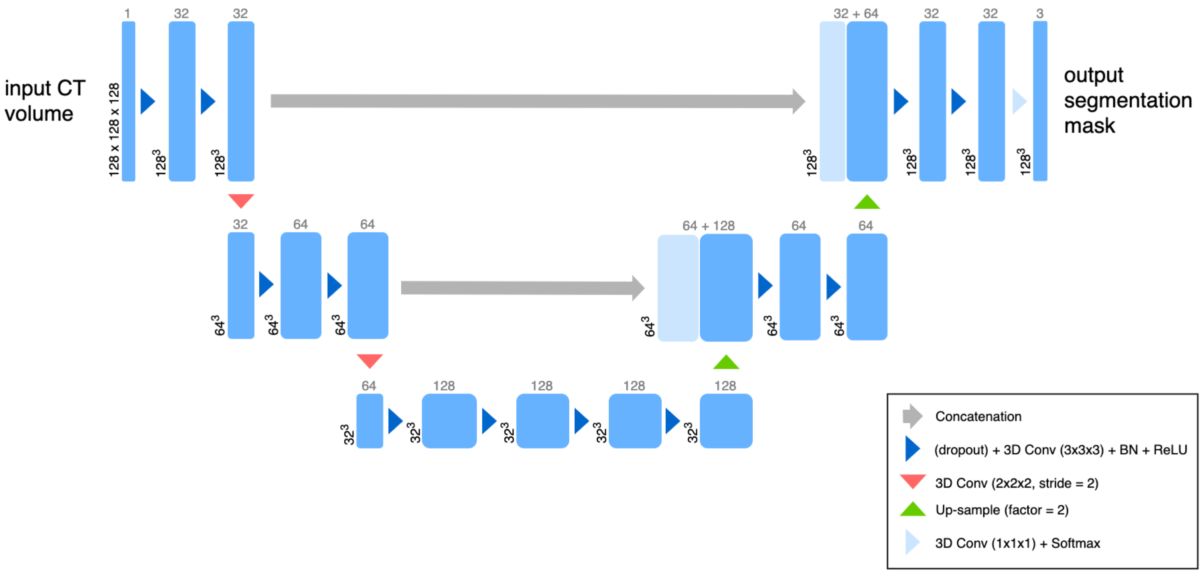

Supplementary Figure 9. A reduced 3D U-Net architecture. The U-Net is a convolutional "encoder-decoder" model for semantic segmentation of 2D and 3D images[1,2]. In this simplified model, convolutional layers with a stride of 2 are used for down-sampling, while the up-sampling operation was performed with the nearest neighbor algorithm. Here, convolutions use filter sizes of 3x3x3, dropout is applied to every convolutional layer, and the softmax function is used on the last layer to produce class pseudo-probabilities. Blue boxes correspond to 3D multi-channel feature maps, with the number of channels denoted on top, and the size of the spatial dimensions marked in the lower left.

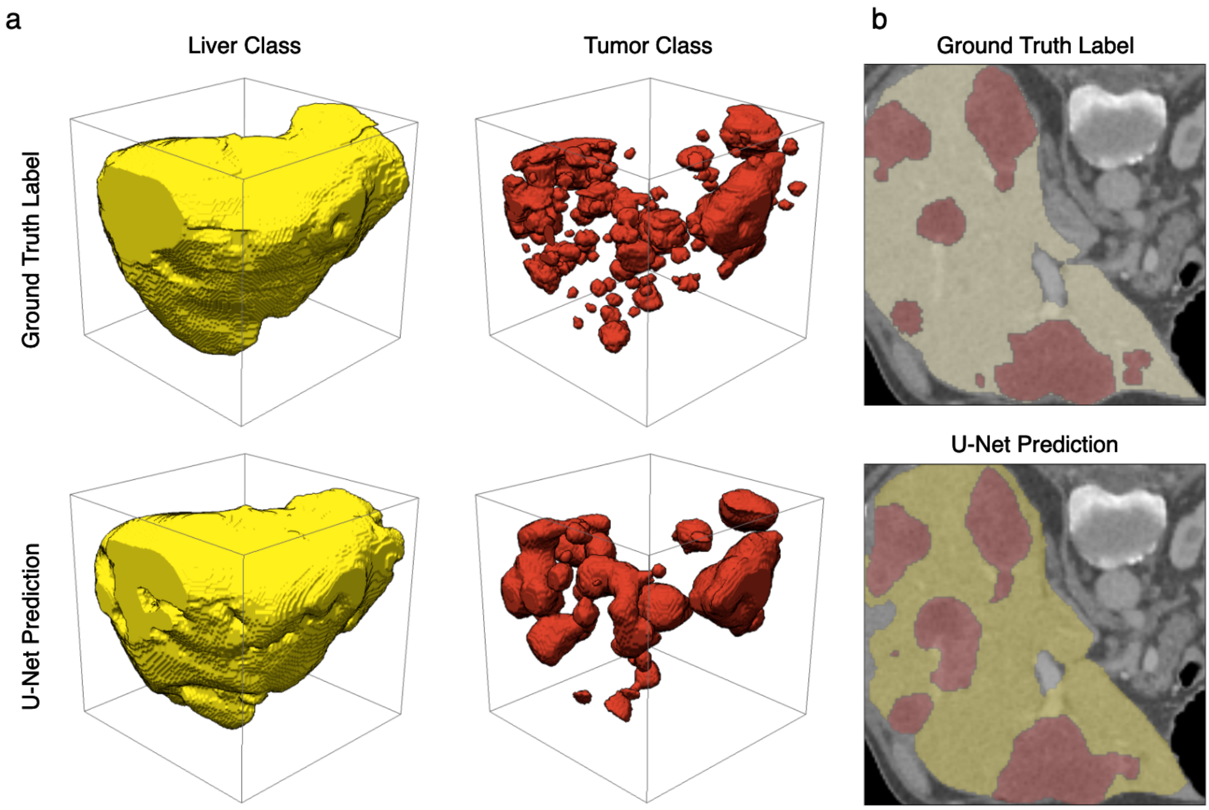

Supplementary Figure 10. Prediction results example of a deterministically trained U-Net model on the LiTS dataset. (a) Volumetric renderings of ground truth labels for the liver class (yellow) and tumor class (red) voxels of a tomogram from the test set, with the corresponding predictions from the deterministically trained model (lower row). (b) Cross-section of the same tomogram, with ground truth labels and model predictions (liver class in yellow, tumor class in red).

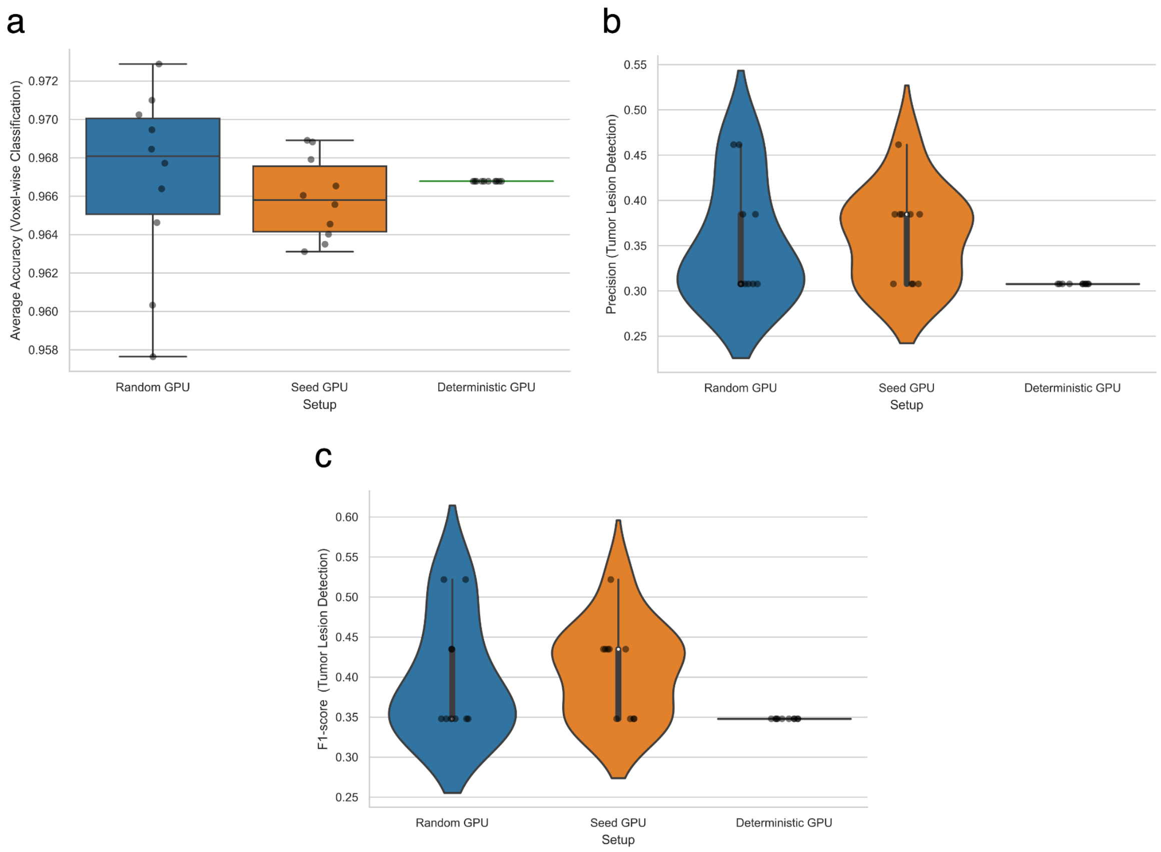

Supplementary Figure 11. Semantic segmentation and tumor lesion detection metrics for prediction results from the reduced 3D U-Net model trained on the LiTS dataset. (a) Boxplot of multi-class segmentation accuracy, measured as the averaged voxel-wise classification accuracy over the testing dataset. (b) Distribution of precision values for tumor lesion detection at an IoU threshold of 0.2 for all trained models. (c) Distribution of F1-scores for tumor lesion detection using the same settings.

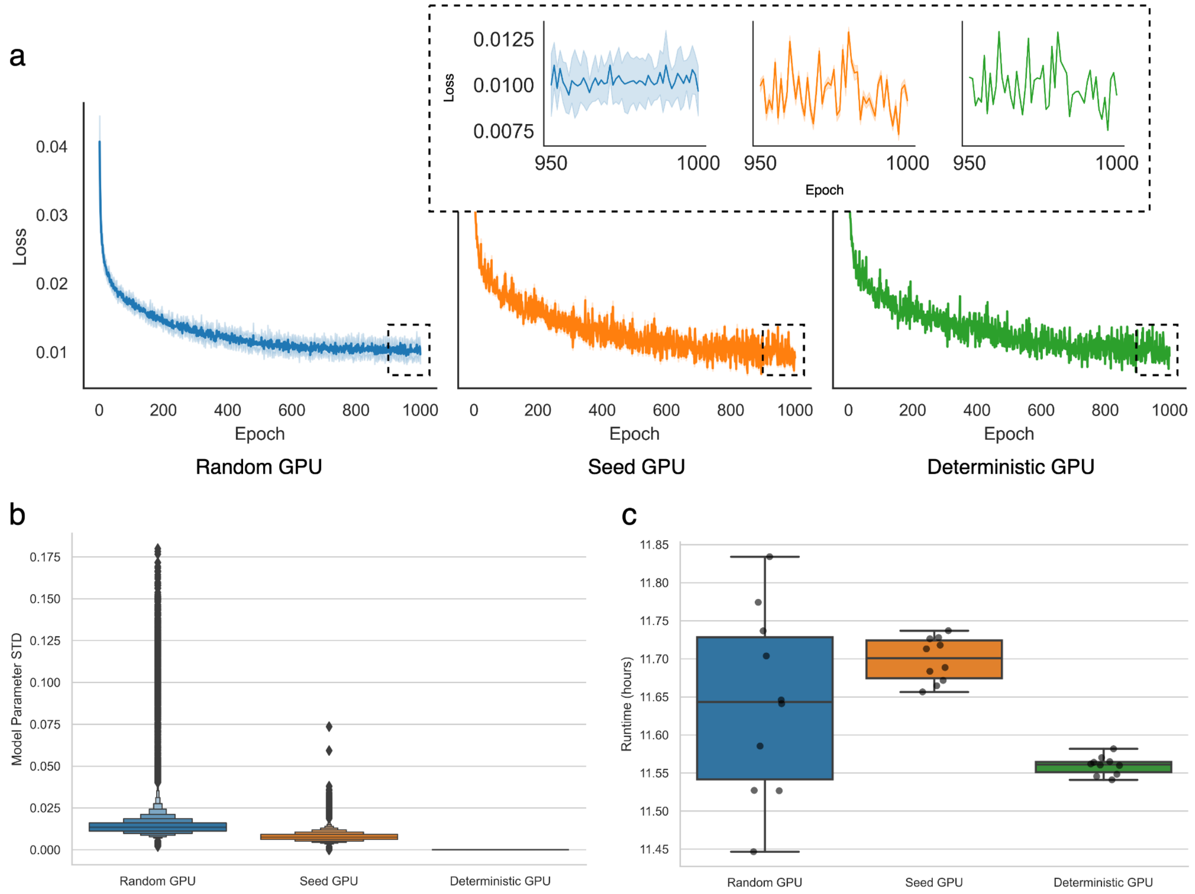

Supplementary Figure 12. Epoch-wise differences in loss between training runs (N=10) in different experimental settings (Random, Seed, Deterministic) for the U-Net model trained on the LiTS dataset, and deviation of model parameters for each of the settings. (a) Loss value during training is shown for different experimental settings, the standard deviation between separate runs is indicated with error bands. The dashed lines mark the zoomed in regions of the curves, corresponding to the last 50 epochs. (b) Letter-value plot of standard deviation values of U-Net model parameters (weights and biases) across training runs (resulting models), the standard deviation of all 3,682,595 parameters was calculated for all experimental settings. (c) Training runtimes (1000 epochs on a model with 3,682,595 trainable parameters) for runs of all experimental settings. All training runs were computed using 2 GPUs, each an NVidia Tesla V100 (32 GB).

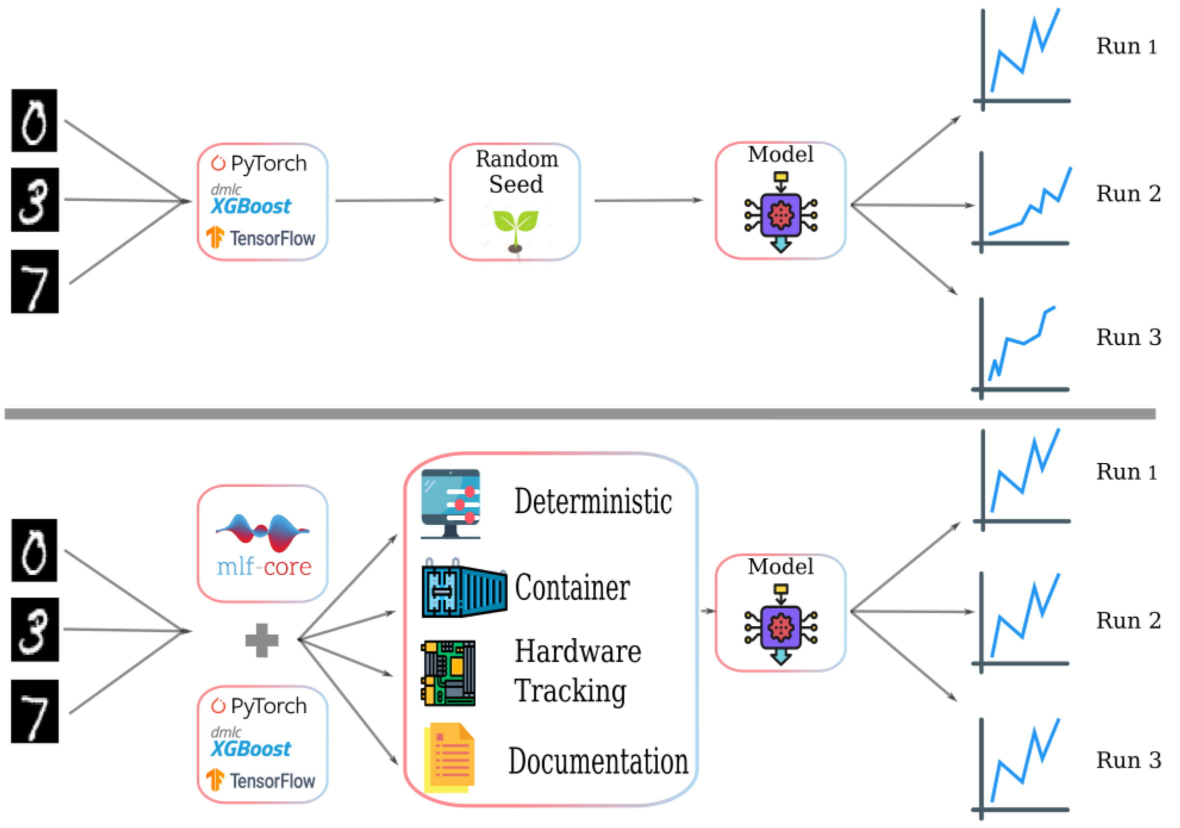

Supplementary Figure 13. Training models with common machine learning libraries with all random seeds set leads to non-deterministic behavior (top). Contrary, training a model with mlf-core ensures that all required random seeds are set and deterministic algorithms are forced with the mlf-core linter, the runtime environment is containerized by leveraging Conda and Docker containers, the hardware is tracked using system-intelligence and the model is fully documented with a Sphinx documentation setup together with the full training history as recorded with MLflow, leading to fully reproducible machine learning (bottom).

Supplementary Methods

PyTorch determinism evaluation setup

To test the deterministic settings of the PyTorch library, a neural network with 2 convolutional (32 and 64 neurons), 2 fully connected (128 and 10 neurons), and 2 dropout (0.25) layers was used. The respective activation functions were rectified linear functions followed by 2-dimensional max-pooling after the second convolutional layer. Optimization was conducted using the Adam optimizer to minimize a log-likelihood loss. Hyperparameters were left at their default values. The models were trained for 25 epochs with default weight initialization. The models were trained to classify images of the MNIST[3] dataset, which contains 70000 (60000 training and 10000 testing) handwritten 28 times 28 greyscale digits, into the correct digit.

All PyTorch experiments were conducted with PyTorch version 1.7.1 and multi-GPU support was enabled using the *DataParallel* API.

To evaluate determinism on the PyTorch library, three different experimental setups were employed:

- *Random*: no random seeds were set.
- Seeds: Random seeds were set for NumPy, PyTorch, Python's Random module and the Python hash seed.
- Deterministic: All random seeds of the setup *seeds* were set together with the enforcement of all deterministic algorithms and cuDNN benchmark was disabled (Supplementary Figure 1).

Further, all functions labeled as knowingly non-deterministic by PyTorch (https://pytorch.org/docs/stable/notes/randomness.html) were explicitly avoided.

The metrics used for determinism evaluation were the negative log-likelihood training loss after the final training epoch and the image classification accuracy. Both are also available online:

https://github.com/mlf-core/machine_learning_determinism_evaluation/blob/master/results/pytorch.csv

TensorFlow determinism evaluation setup

To evaluate the determinism for models built with the TensorFlow library, the same model and training data described for the PyTorch library were used. The TensorFlow experiments were conducted using TensorFlow version 2.2 with multi GPU support provided by the *tf.distribute.MirroredStrategy()* function.

To evaluate determinism on the TensorFlow library, three different setups were employed:

- *Random*: No seeds were set.
- *Seeds*: Random seeds were set for NumPy, TensorFlow, Python's Random module and the Python hash seed.
- *Deterministic*: All random seeds of the setup *seeds* were set together with the *TF_DETERMINISTIC_OPS* environment variable (Supplementary Figure 1).

Moreover, all non-deterministic functions identified by NVIDIA (https://github.com/NVIDIA/framework-determinism) in TensorFlow 2 were avoided.

The metrics used for determinism evaluation were the cross entropy training loss after the final training epoch and the image classification accuracy. Both are also available online: https://github.com/mlf-core/machine_learning_determinism_evaluation/blob/master/results/tensorflow.csv

XGBoost determinism evaluation setup

The forest cover type using the Covertype dataset[4], was used to evaluate determinism for the XGBoost library.XGBoost models were trained with the hyperparameter *subsample* (subsample ratio of the training instances) of 0.5, *colsample_bytree* (subsample ratio of columns) of 0.5 and *colsample_bylevel* (subsample ratio of columns for each level) of 0.5 to introduce random factors. Additionally, some experiments used the parameter *single_precision*, which reduces the precision when building histograms from double to single. This usually leads to faster convergence at the cost of precision.

Prior to XGBoost 1.1.0 the *hist* histogram building algorithm and its GPU accelerated counterpart *gpu_hist* were susceptible to accumulations of floating-point errors (https://github.com/dmlc/xgboost/issues/5632). Later releases feature deterministic histogram building through floating-point pre-rounding techniques (https://github.com/dmlc/xgboost/pull/5361). XGBoost models were either trained with the officially distributed 1.0.2 version, a CUDA 9 compiled 1.1.0 version or a CUDA 10

compiled 1.1.0 version. Multi GPU support was provided by a *LocalCUDACluster* with Dask (https://dask.org) version 2.14.0, Dask Cuda version 0.13.0 and Dask-ML version 1.4.0.

Hence, the following setups were used to evaluate determinism:

- *No seeds*: Since XGBoost uses a default seed of 0 a randomly picked seed was set for XGBoost.
- Seeds: random seeds were set for Numpy, XGBoost, the XGBoost model's seed, Python's random module and the Python hash seed.
- Single-precision: *single_precision_histogram* was enabled (Supplementary Figure 1).

XGBoost's *allreduce* operations were avoided since they have not yet been verified to run deterministically (https://github.com/dmlc/xgboost/issues/5023).

The metrics used for determinism evaluation was the multiclass classification error rate. The results are also available online:

https://github.com/mlf-core/machine_learning_determinism_evaluation/blob/master/results/xgboost.csv

Evaluating machine learning library determinism

All deterministic machine learning experiments were conducted with Nextflow version 20.01.0 build 5264 on the hardware systems shown in Supplementary Table 1. Every experiment was conducted with Docker containers with a custom base inheriting from *nvidia/cuda:10.2-base-ubuntu18.04*. The full setup including Docker containers, Conda environments and code are available on GitHub: https://github.com/mlf-core/machine_learning_determinism_evaluation. The execution time of all trained models was calculated with Python's inbuilt *timeit* function.

Analysis of scRNA-seq data with TensorFlow

Analysis of scRNA-seq data was performed using the Python Scanpy library[5] (v1.6.0) and the 3k PBMCs from a Healthy Donor dataset available from 10X Genomics (https://support.10xgenomics.com/single-cell-gene-expression/datasets/1.1.0/pbmc3k). The data was downloaded using the Scanpy function *sc.datasets.pbmc3k()*. Cells with less than 200 genes and genes found in less than 3 cells were removed. Counts were normalized to library size (1000 counts per cell) and transformed to logarithmic space. Finally, the count

matrix was subsetted to highly variable genes and scaled to zero mean and maximum values of 3 using Scanpy-inbuilt functions.

A simple autoencoder model with layer sizes [256, 128, 64, 32, 64, 128, 256] was fitted to the data. Optimization was performed using the Adam algorithm and a mean squared error loss, a fixed learning rate of 0.001 and a batch size of 256. We fixed the training to 1000 epochs per run.

For comparison of different training settings, we compared the loss per epoch between runs. UMAP embedding was done by extracting the encoding stored in the 32-units layer of the autoencoder and applying the UMAP algorithm[6]. Similarly, for clustering, we extracted the latent space embedding and used the Scanpy functions *sc.pp.neighbors()* and *sc.tl.leiden()* to generate clusters with the Leiden algorithm[7]. To evaluate how differences in the embedding affect cell clustering, we compared the cluster sizes between runs, as cells assigned to different clusters at different runs will lead to cluster size differences. The model is openly available under https://github.com/mlf-core/sc-autoencoder.

Use case XGBoost - Liver cancer data model

To train the XGBoost model, gene expression data was collected from 374 hepatocellular carcinoma samples in the Cancer Genome Atlas Liver Hepatocellular Carcinoma (TCGA-LIHC)[8] cohort, including 50 healthy liver samples from the same project; and 136 healthy liver samples of the Genotype-Tissue Expression (GTEx) project[9]. Gene count data of both projects was obtained through the recount2 project portal (https://jhubiostatistics.shinyapps.io/recount/) [10].

The gene counts were transformed into transcript per million (TPM) values using the median transcript length for each gene, which was determined using GTFtools[11].

The gene features used to train the model were reduced to the 556 genes present in the Kyoto Encyclopedia of Genes and Genomes (KEGG) pathway hsa05200 with the title "Pathways in cancer - Homo sapiens (human)"[12]. The data was split into a 75% training and a 25% testing subset.

An XGBoost classification model was fitted to the dataset, and trained for 1000 epochs. The other hyperparameters of the model were *objective: binary:logistic, colsample_bytree: 0.6, learning_rate: 0.2, max_depth: 3, min_child_weight: 1, subsample: 0.7*. Unlisted parameters were left at their default values. The model's hyperparameters were selected following an

exhaustive grid search, in which the best hyperparameters were selected based on the highest Matthews correlation coefficient (MCC) value in a 5-fold cross-validation on the training dataset. The model is openly available under https://github.com/mlf-core/lcep. To evaluate determinism, we compared the differences in feature weights between runs, which are a measure of the importance of each feature in the input data for the model's predictions. The order of the mean feature weights of the non-deterministic run was compared with the order of features of a deterministic setting.

Liver tumor semantic segmentation with PyTorch

This semantic segmentation analysis was based on the training dataset of the *Liver Tumor Segmentation Benchmark* (LiTS)[13], consisting of 131 abdominal CT scans of patients with HCC, and the corresponding ground-truth segmentation masks of liver and tumor lesions, as annotated by trained radiologists. The tomograms are a mix of pre- and post-therapy abdomen scans, from different CT scanners, using different acquisition protocols, and with a varied number and size of tumors. The segmentation masks are volumetric images where each voxel has a value denoting a class label (0 for background, 1 for liver tissue, and 2 for tumor). The annotated dataset can be downloaded from *codalab* (https://competitions.codalab.org/competitions/17094), or accessed via torrent (https://academictorrents.com/details/27772adef6f563a1ecc0ae19a528b956e6c803ce).

The provided 131 tomograms and segmentation masks with image sizes of 512x512 in the X and Y dimensions, and a variable size in Z (74 - 987), were curated to satisfy memory restrictions. They were first down-scaled by a factor of 2 (to resolutions ranging from 1.12 to 2.0 mm in X and Y, and 0.9 to 12.0 mm on Z) using the *skimage.transform.downscale_local_mean* function of the Scikit-image Python library (https://scikit-image.org/), down-sampling was performed using local averaging. Subsequently, volumes of size 128x128x128 located on the geometric center of the liver annotation, in both the tomograms and segmentation masks, were extracted to create the analyzed dataset.

We used a 3D U-Net architecture[2] (considering 1 input channel and 3 classes), inspired by the reduced model employed by DeepFinder[14]. Accordingly, our model has only two

down-sampling stages and contains supplementary convolutional layers in the lowest stage to consider a large spatial context (Supplementary Figure 8). Additionally, we used ReLUs as activation functions and trained the models for 1000 epochs using the ADAM algorithm[15], with a learning rate of 0.0001 and weight decay of 0.0001. We set the batch size to 4 and used dropout with a rate of 0.25. A focal loss[16] was used with an alpha vector (class weights) of (0.2, 1.0, 2.5) and a gamma value of 2.
After being revealed as non-deterministic by the mlf-core linter, we modified our U-Net model to use convolutional layers with a stride of 2 for down-sampling, while the up-sampling operation was performed with the nearest neighbor algorithm. These operations are usually implemented using max-pooling layers and up-convolutions (transposed-convolution), respectively. These modifications were necessary since the used PyTorch library (version 1.7.1) does not offer deterministic implementations of the *torch.nn.MaxPool3d* and *torch.nn.ConvTranspose3d* operations. The model is openly available under https://github.com/mlf-core/liver-ct-segmentation.

To evaluate reproducibility we compared the loss values during training runs. We also compared segmentation predictions by measuring performance on a small test set (10% of the available data, 13 randomly selected tomograms), using the Jaccard index or *Intersection over Union* (IoU) as a similarity metric (against expert ground-truth labels), for all training settings. Additionally, we calculated the standard deviation of voxel-wise softmax values, as produced by the models. Finally, we calculated the tumor burden for each tomogram in the test set, and measured the standard deviation among experiment runs.

We presented performance metrics to evaluate this semantic segmentation task, specifically the direct output of the U-Net model (i.e. a mask with pixel-wise classification predictions for 3 classes), and then evaluated tumor lesion detection metrics, as described by the LiTS[40] (ref). We used the mean of the Jaccard index to report segmentation performance, it measures the amount of overlap between the predicted and ground-truth segmentations. *IoU* can be described as follows:

$$IoU\,(A,\,B) \;=\; \frac{|\,A \cap B\,|}{|\,A \cup B\,|} \qquad (1)$$

Where A and B are class-specific binary masks. Additionally, we calculated the multi-class, voxel-wise classification accuracy, averaged over the testing dataset, this segmentation metric was calculated as follows:

$$Accuracy = \frac{correct\ voxel\ classifications}{all\ classifications} \quad (2)$$

To perform direct assessment of tumor lesion detection, we first evaluate the precision of lesion detection at an *IoU* threshold of 0.2. That is, if the predicted tumor mask has sufficient overlap with its corresponding reference lesion, then the lesion is considered as predicted. This approach is often used to measure detection metrics from segmentation results[40], it allows the count of true positives (TP), false positives (FP), and false negatives (FN). Here a low *IoU* threshold is used to increase detection sensitivity. Detection metrics for precision, recall, and the F1-score (i.e. measure of the harmonic mean from precision and recall) were calculated as follows:

$$precision = \frac{TP}{TP + FP} \quad (3)$$

$$recall = \frac{TP}{TP + FN} \quad (4)$$

$$F1 = \frac{2}{precision^{-1} + recall^{-1}} \quad (5)$$

References

1. Ronneberger, O., Fischer, P. & Brox, T. U-Net: Convolutional Networks for Biomedical Image Segmentation. in *Medical Image Computing and Computer-Assisted Intervention – MICCAI 2015* 234–241 (Springer International Publishing, 2015).

2. Çiçek, Ö., Abdulkadir, A., Lienkamp, S. S., Brox, T. & Ronneberger, O. 3D U-Net: Learning Dense Volumetric Segmentation from Sparse Annotation. in *Medical Image Computing and*

Computer-Assisted Intervention – MICCAI 2016 424–432 (Springer International Publishing, 2016).

3. Lecun, Y. THE MNIST DATABASE of handwritten digits. *http://yann. lecun. com/exdb/mnist/*.
4. Dua, D. & Graff, C. UCI Machine Learning Repository. (2017).
5. Wolf, F. A., Angerer, P. & Theis, F. J. SCANPY: large-scale single-cell gene expression data analysis. *Genome Biol.* **19**, 15 (2018).
6. McInnes, L., Healy, J. & Melville, J. UMAP: Uniform Manifold Approximation and Projection for Dimension Reduction. (2018).
7. Traag, V. A., Waltman, L. & van Eck, N. J. From Louvain to Leiden: guaranteeing well-connected communities. *Sci. Rep.* **9**, 5233 (2019).
8. TCGA-LIHC - the cancer imaging archive (TCIA) public access - cancer imaging archive wiki. https://wiki.cancerimagingarchive.net/display/Public/TCGA-LIHC.
9. GTEx Consortium. Human genomics. The Genotype-Tissue Expression (GTEx) pilot analysis: multitissue gene regulation in humans. *Science* **348**, 648–660 (2015).
10. Collado-Torres, L. *et al.* Reproducible RNA-seq analysis using recount2. *Nat. Biotechnol.* **35**, 319–321 (2017).
11. Li, H.-D. GTFtools: a Python package for analyzing various modes of gene models. *Cold Spring Harbor Laboratory* 263517 (2018) doi:10.1101/263517.
12. Kanehisa, M. & Goto, S. KEGG: kyoto encyclopedia of genes and genomes. *Nucleic Acids Res.* **28**, 27–30 (2000).
13. Bilic, P. *et al.* The Liver Tumor Segmentation Benchmark (LiTS). *arXiv [cs.CV]* (2019).
14. Moebel, E. *et al.* Deep learning improves macromolecule identification in 3D cellular cryo-electron tomograms. *Nat. Methods* **18**, 1386–1394 (2021).
15. Kingma, D. P. & Ba, J. Adam: A Method for Stochastic Optimization. (2014).
16. Lin, T.-Y., Goyal, P., Girshick, R., He, K. & Dollar, P. Focal Loss for Dense Object Detection. *IEEE Trans. Pattern Anal. Mach. Intell.* **42**, 318–327 (2020).
